# Magnolol dimer-derived fragments as PPARγ-selective probes†
†Electronic supplementary information (ESI) available. See DOI: 10.1039/c8ob01745j


**DOI:** 10.1039/c8ob01745j

**Published:** 2018-09-20

**Authors:** Dominik Dreier, Mirta Resetar, Veronika Temml, Lukas Rycek, Nicolas Kratena, Michael Schnürch, Daniela Schuster, Verena M. Dirsch, Marko D. Mihovilovic

**Affiliations:** a Institute of Applied Synthetic Chemistry , TU Wien , Getreidemarkt 9/163 , A-1060 Vienna , Austria . Email: marko.mihovilovic@tuwien.ac.at; b Department of Pharmacognosy , University of Vienna , Althanstraße 14 , 1090 Vienna , Austria; c Institute of Pharmacy/Pharmacognosy and Center for Molecular Biosciences Innsbruck , University of Innsbruck , Innrain 80/82 , 6020 Innsbruck , Austria; d Institute of Pharmacy/Pharmaceutical Chemistry and Center for Molecular Biosciences Innsbruck , University of Innsbruck , Innrain 80/82 , 6020 Innsbruck , Austria; e Paracelsus Medical University Salzburg , Institute of Pharmacy , Department of Pharmaceutical and Medicinal Chemistry , Strubergasse 21 , 5020 Salzburg , Austria

## Abstract

Partial agonists of the transcription factor PPARγ (peroxisome proliferator-activated receptor γ) have shown potential for the treatment of metabolic and inflammatory conditions and novel activators serve as valuable tool and lead compounds. Based on the natural product magnolol (**I**) and recent structural information of the ligand–target interaction we have previously developed magnolol dimer (**II**) which has been shown to have enhanced affinity towards PPARγ and improved selectivity over RXRα (retinoid X receptor α), PPARγ's heterodimerization partner. In this contribution we report the synthesis and evaluation of three fragments of the dimeric lead compound by structural simplifications. Sesqui magnolol A and B (**III** and **IV**) were found to exhibit comparable activities to magnolol dimer (**II**) and selectivity over RXRα persisted. Computational studies suggest a common pharmacophore of the distinctive biphenyl motifs. Truncated magnolol dimer (**V**) on the other hand does not share this feature and was found to act as an antagonist.

## Abstract 2

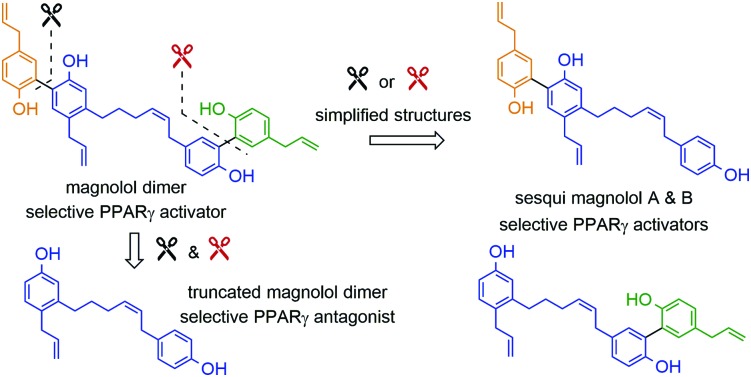
Sesqui magnolol A & B have been found to be selective partial PPARγ agonists while truncated magnolol dimer acts as an antagonist.

## Introduction

**Fig. 1:**
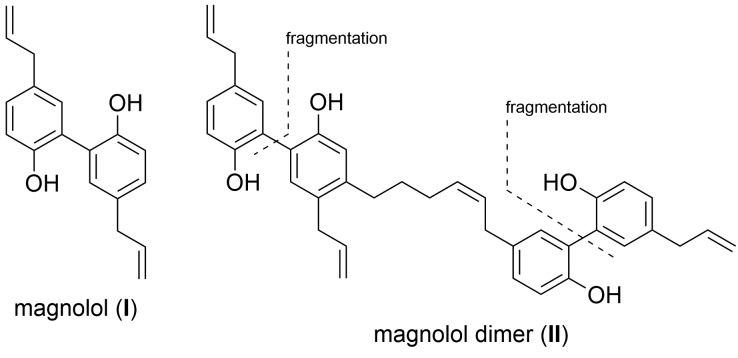
Structure of PPARγ agonists magnolol (**I**) and magnolol dimer (**II**).

PPARs (Peroxisome Proliferator-Activated Receptors) are members of the nuclear receptor family of transcription factors. They require ligand-binding and heterodimerization with the nuclear receptor RXR (Retinoid X Receptor) to activate gene transcription. PPARγ has an established role in adipocyte differentiation and function, lipid and glucose metabolism, and macrophage activation.^1^,^2^ PPARγ agonists have been proposed to exhibit beneficial effects on treating some forms of cancer, metabolic and inflammatory conditions.^3^ The only clinically available PPARγ-targeting drugs, TZDs (thiazolidinediones, *e.g.* troglitazone, rosiglitazone and pioglitazone) have been used to treat type 2 diabetes due to their insulin sensitizing and anti-hyperlipidemic effects.^4^ Unfortunately, TZD administration was accompanied by side effects such as weight gain, fluid retention, bone fractures and their usage was later restricted due to hepatotoxicity and heart failure concerns.^3^,^5^ The proven beneficial agonistic effects of PPARγ call for development of new ligands, but the challenge remains to develop sufficiently efficacious ligands that would act in a target- and tissue-specific manner. Current efforts are directed towards developing selective PPAR agonists, which would activate desired physiological responses in tissues of interest, exclusively. It has been shown that different partial agonists cause distinct conformational changes that lead to different coactivator recruitment and activation of discrete sets of genes.^3^ In this work, we report a rational design approach and synthesis towards new synthetic ligands, derived from the structure of the known PPARγ agonist magnolol (**I**) and magnolol dimer (**II**), developed previously in our laboratory, employing a modified fragment approach (Fig. 1).^6^

Magnolol (**I**) is a lignan found in the bark of the plant *Magnolia officinalis* which has been used in traditional Chinese medicine as potential anti-inflammatory, anti-cancer, and neuromodulatory agent as well as a lead structure for synthetic derivatives.^7^,^8^ We previously demonstrated that magnonol (**I**) is a partial PPARγ agonist.^9^ However, magnolol (**I**) was found to activate RXRα as well.^10^ Even though RXR agonists themselves can activate PPAR:RXR heterodimers, given that they also activate other permissive heterodimers, and can thus elicit unwanted responses, we aimed to develop PPARγ-specific ligands. In order to do so, we utilized a particular feature of the PPARγ ligand binding pocket – its size, which is substantially larger than in other nuclear receptors.^11^ Fakhrudin *et al.* have predicted, which was later confirmed by crystallography, that two magnolol molecules bind into the PPARγ ligand binding site.^9^,^10^ Within a trans-disciplinary project aiming at the exploitation of bioactive natural compounds from plants^12^ as starting point for the design of pharmacological probes,^13^ we became interested in this particular binding mode of magnolol at the target receptor. Thus, in our previous work, we computationally designed and synthesized magnolol dimer (**II**) inspired by the dual occupation of the binding site. Magnolol dimer (**II**) was shown to bind to the PPARγ ligand binding pocket, but not to the one of RXRα.^14^ Using reporter gene assays, we demonstrated that magnolol dimer (**II**) can specifically transactivate PPARγ. In the current contribution, we further developed three novel magnolol dimer-based ligands following a reversed fragment based approach. By simplifying the dimeric structure upon “cutting” various rings, we aimed to investigate the influence of the individual aryl moieties on the resulting activity. “Sesqui” magnolol A (**III**) and sesqui magnolol B (**IV**) were found to display PPARγ partial agonism while truncated magnolol dimer (**V**) can act as a PAPRγ antagonist. Here, we report the synthesis, biological evaluation and *in silico* binding of these compounds, and propose them to be interesting candidates for further evaluation.

## Results and discussion

### Retrosynthetic analysis

Sesqui magnolol A (**III**) and sesqui magnolol B (**IV**) were envisaged by omitting one of the outer phenol rings of magnolol dimer (**II**). Truncated magnolol dimer (**V**) results from removing both external phenol domains. These three novel fragments of magnolol dimer (**II**) should provide valuable insight into the binding behaviour of this compound class.

**Scheme 1:**
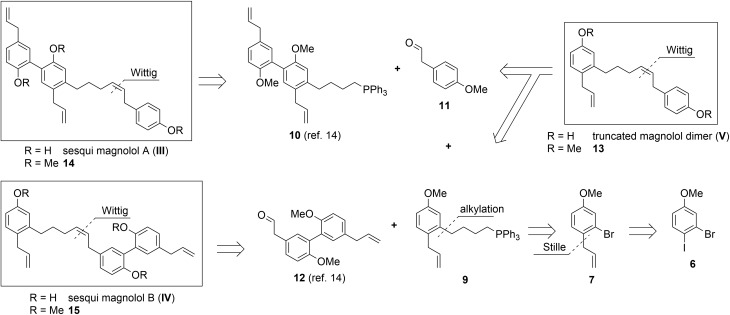
Retrosynthetic analysis of target compounds **III**, **IV** and **V**.

The rationale for the synthesis of the sesqui target compounds **III** and **IV** is in line with the reported synthetic route towards magnolol dimer (**II**) and is depicted in Scheme 1. The phenol groups have to remain protected throughout the synthesis. A very robust protecting group like methyl was thus chosen and global deprotection was planned to be the last step. The two aromatic systems were joined in a Wittig olefination; using a non-stabilized alkyl ylide, *e.g.***9**, to secure the desired (*Z*)-stereochemistry of the formed double bond. The alkyl chain in **9** might be introduced by lithiation/Grignard formation of aryl bromide **7** and subsequent reaction with desired alkyl electrophile. 2-Bromo-estragole (**7**) can be obtained by Stille cross-coupling of 2-bromo-1-iodo-4-methoxybenzene (**6**) with an allylstannane. The iodide **6** is formed by iodination of commercially available 3-bromoanisole (**5**). The truncated magnolol dimer (**V**) can likewise be prepared by Wittig reaction of aldehyde **11** with **9** followed by methyl ether deprotection.

### Synthesis

**Scheme 2:**
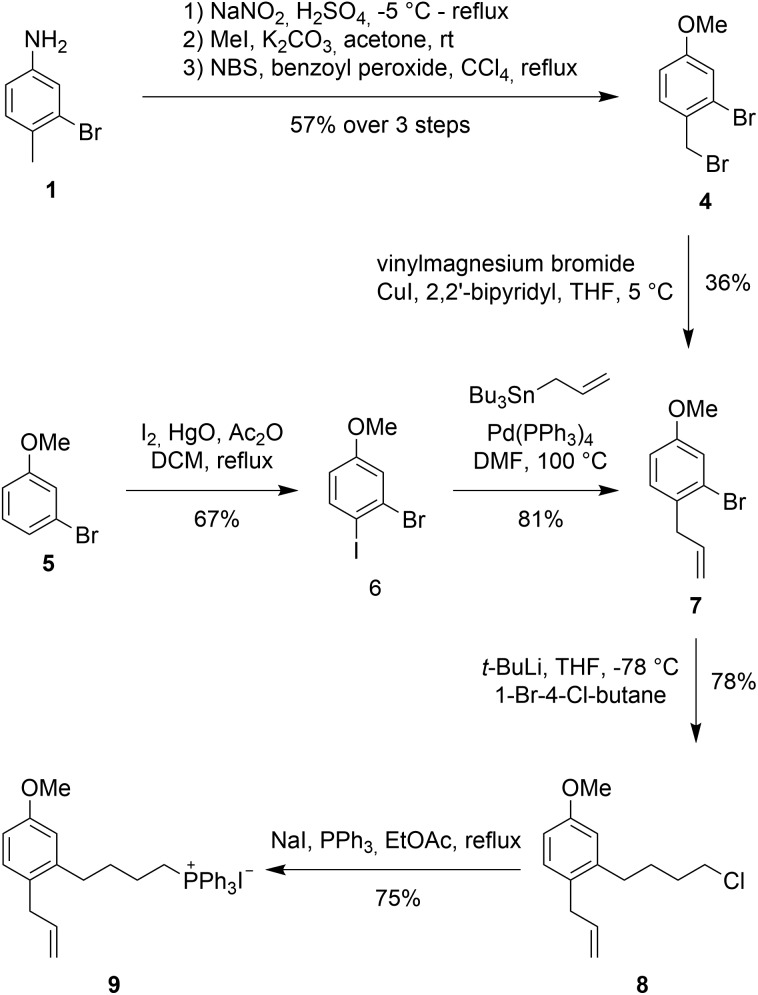
Synthesis of Wittig salt **9**.

**Scheme 3:**
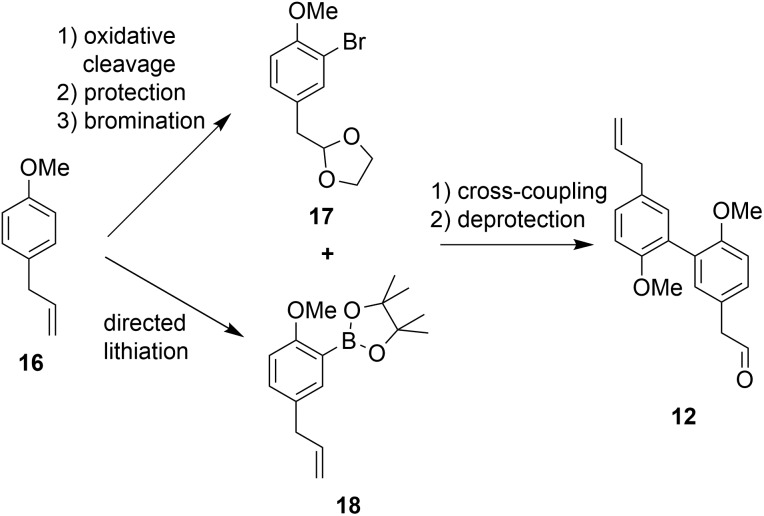
Reported synthesis of building block **12**.

An initial approach towards **9** started from 3-bromo-4-methylaniline **1** (see Scheme 2, **2** and **3** not shown), which was converted to 2-bromo-1-(bromomethyl)-4-methoxybenzene (**4**) *via* functional group interconversion, methyl protection, and bromination. Next, dibromide **4** was subjected to Kumada coupling with vinyl magnesium bromide.^15^,^16^ Unfortunately, the aryl bromide can undergo facile metal–halogen-exchange under these conditions and formation of the corresponding biaryl dimer and other side-products were observed in considerable quantities. The desired product **7** could be isolated as a 3 : 1 mixture of products in 36% yield.

An improved access to **7** started from 3-bromoanisole (**5**). Iodination of the aryl system on electronically activated C-2 and C-6 should be suppressed by the steric influences of the methoxy and bromine substituent. Indeed, iodination using *in situ* formed diiodine monoxide (from HgO and I_2_)^17^,^18^ proceeded to give 2-bromo-1-iodo-4-methoxybenzene (**6**) in good yield. Less than 10% of the undesired *ortho*-iodo isomers were formed during this reaction. Employing a Stille coupling with simple tetrakis(triphenylphosphine)palladium in DMF at 100 °C gave allylbenzene **7** after just 30 minutes in 81% yield.

For the introduction of the alkyl side chain we were inspired by a publication by Back *et al.*^19^ Lithium halogen exchange of the bromo group in **7** was achieved with 2 equivalents of *t*-BuLi at –78 °C in THF. Addition of 1-bromo-4-chlorobutane at –78 °C resulted in the desired transformation providing butylchloride **8** as the product in 78% yield.

With alkyl chloride **8** in hand, Wittig salt formation was evaluated. The formation of the phosphonium salt did not proceed directly from the chloride, instead a Finkelstein reaction was required for activation to convert chloride **8** into the corresponding iodide (86% yield). Subsequent substitution with triphenylphosphine proceeded quantitatively. Both transformations could also be performed in a one-pot fashion in EtOAc and delivered the key fragment **9** in 75% yield after recrystallization.

**Scheme 4:**
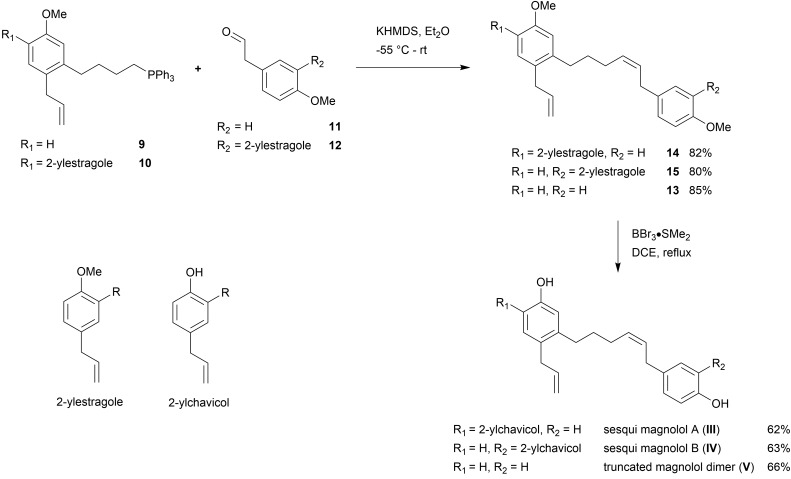
Wittig reaction and deprotection.

The second building block for the synthesis of sesqui magnolol B (**IV**) was represented by diaryl aldehyde **12**. It was prepared starting from commercially available estragole (**16**) using a sequence published in earlier work^14^ (overview see Scheme 3). The first step (oxidative cleavage) gives rise to the second required aldehyde group in **11** (Scheme 1). These 2-phenylacetaldehydes were prone to auto-oxidation as well as degradation to the corresponding benzaldehydes^20^ and were protected, consequently. Additionally, special conditions were necessary during the coupling of **17** and **18** to prevent double-bond isomerization to the corresponding styrene derivatives.^21^,^22^ With all necessary fragments available the synthesis of biological probes could commence. Wittig salt **9** was reacted with aldehydes **11** and **12** giving products **13** and **15**, respectively. Diaryl Wittig salt (**10**) from earlier work^14^ was reacted with aldehyde **11** furnishing protected sesqui magnolol A (**14**). Reactions were performed in Et_2_O using KHMDS as a strong base. Interestingly, upon scale-up this reaction displayed significant olefin isomerization to a number of possible styrene derivatives, which did not occur at a smaller scale. (*Z*)-Isomers were obtained exclusively in all instances and in good yields (see Scheme 4). Finally, global methoxy deprotection was attained by treatment with BBr_3_·SMe_2_ in boiling 1,2-DCE. The di/triphenols **III**, **IV** and **V** were isolated as final products in approximately 60% yield by flash column chromatography.

### Biological evaluation

In order to assess the activation potential of the newly synthesized compounds **III**, **IV** and **V** in a cellular assay, we used a one-hybrid luciferase reporter system. In this model, the PPARγ ligand binding domain (PPARγ-LBD) is combined with the DNA-binding domain of the yeast transcription factor Gal4, which then binds to its response element in the promoter of a luciferase reporter gene. Thus, the ligand-induced transcription of the reporter gene with Gal4 hybrids depends only on ligand binding to the PPARγ-LBD, and not on PPAR:RXR heterodimer formation and permissive activation through RXR.

**Fig. 2:**
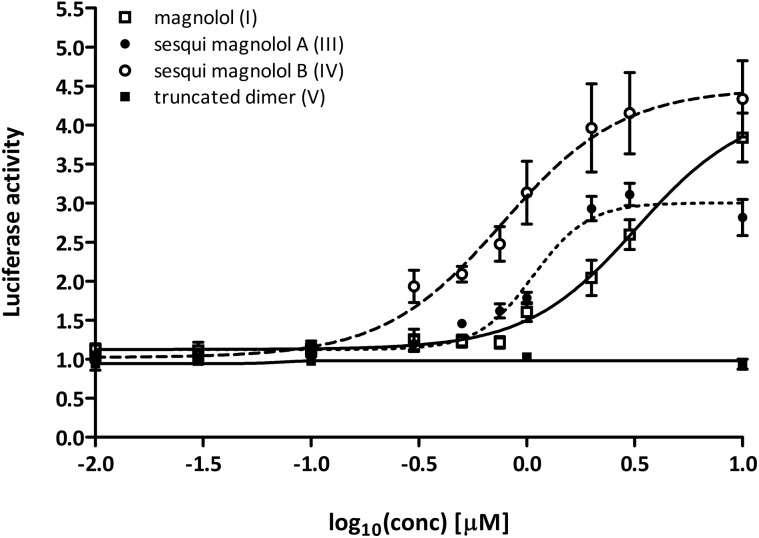
Magnolol (**I**) and magnolol dimer-based derivatives – sesqui magnolol A (**III**) and B (**IV**), but not truncated magnolol dimer (**V**), induced the PPARγ-Gal4 transactivation of the Luciferase gene expression in a dose-dependent manner. Data points represent mean ± SEM. Concentrations were log-transformed and EC_50_ and *E*_max_ values were obtained using the non-linear fitting of the dose–response data using the variable slope.

Sesqui magnolol A (**III**) and sesqui magnolol B (**IV**) were able to activate the PPARγ-Gal4-dependent transcription of the reporter gene in a dose-dependent manner, similar to magnolol (**I**, see Fig. 2). Truncated magnolol dimer (**V**) was unable to induce transactivation. In comparison to magnolol (**I**: EC_50_ = 3.24 μM, 95% CI = 1.96–5.33), the two sesqui compounds **III** and **IV** were found to be more potent binders (**III**: EC_50_ = 1.07 μM, 95% CI = 0.89–1.29, **IV**: EC_50_ = 0.77 μM, 95% CI = 0.51–1.17). In comparison to the full PPARγ agonist pioglitazone (not shown on graph), all compounds showed significantly lower *E*_max_ values (**I**: 4.24 fold, 95% CI = 3.35–5.13; **III**: 3.00 fold, 95%CI = 2.79–3.22, **IV**: 4.47 fold, 95% CI = 3.82–5.13; pioglitazone: 39.38 fold, 95% CI = 37.31–41.46), suggesting they act as partial agonists which has been a particular goal of this project as partial agonist were proposed to lead to fewer side effects.^23^

**Fig. 3:**
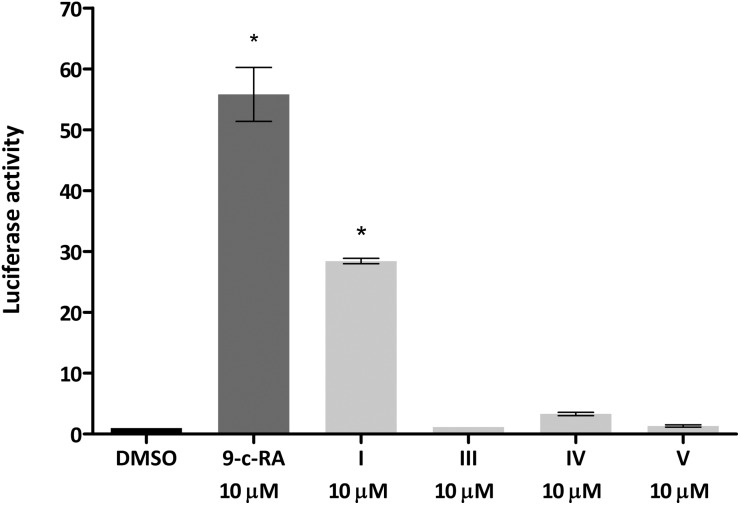
Sesqui magnolol A (**III**), sesqui magnolol B (**IV**) and truncated magnolol dimer (**V**) do not activate the RXRα-Gal4 receptor, in contrast to magnolol (**I**) and a positive control 9-*cis*-retinoic acid. Bars show mean ± SEM. *p < 0.01 in comparison to DMSO, one-way ANOVA with Dunett's *post hoc* test.

We next tested whether the dimer-based derivatives could activate RXRα employing the RXRα-Gal4 hybrid approach. Indeed, none of the dimer-based compounds were able to induce the RXRα-Gal4 transactivation of the Luciferase reporter gene, in contrast to magnolol (**I**, Fig. 3).

Taking the obtained data into account, we hypothesized that sesqui magnolol A (**III**) and sesqui magnolol B (**IV**) bind similarly to PPARγ as they display comparable activation. Although they share different molecular regions with the parent compound magnolol dimer (**II**), one intact biphenyl fragment in **III** and **IV** led to a matching behavior. In consequence, we were intrigued whether the two molecules might share a common pharmacophore where the recognition of the biphenyl fragment is the major contribution to the resulting activity. Concerning the inactivity of truncated magnolol dimer (**V**), we speculated that the molecule's structure is unable to undergo a sufficient number of interactions with the protein due to an amplified simplification of the initial architectures **I** and **II**, respectively.

### Computational assessment

To test our hypothesis we decided to carry out appropriate *in silico* studies. The synthesized compounds sesqui magnolol A (**III**), sesqui magnolol B (**IV**) and truncated magnolol dimer (**V**) were computationally docked into the binding site of PPARγ^10^ to analyze their fit and binding site interactions.

**Fig. 4:**
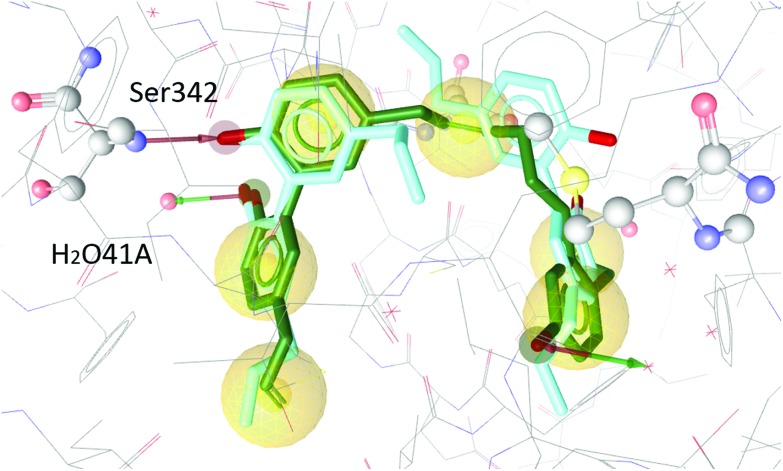
Two magnolol molecules (cyan, A left, B right) co-crystallized in the binding site of PPARγ and the best-ranked docking pose of **IV** (green) and their interactions with the binding site. Green and red arrows signify hydrogen bonds, yellow spheres mark hydrophobic contacts. Interactions with Ser342 and H_2_O41 are predicted.

**Fig. 5:**
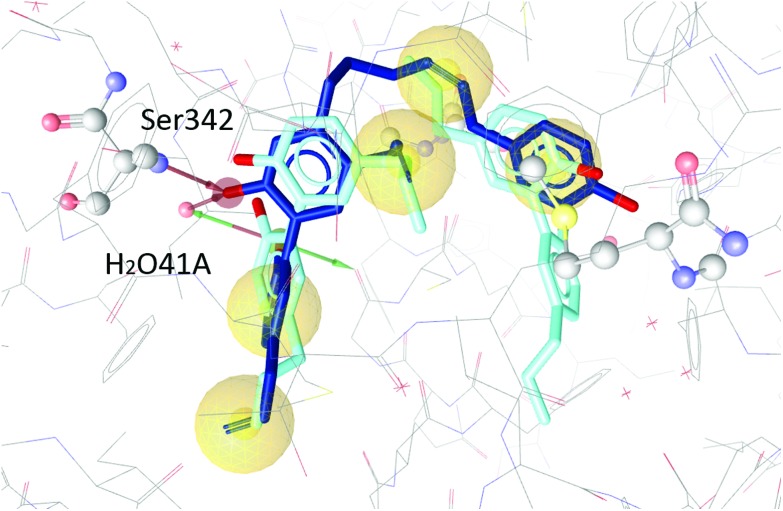
Two magnolol molecules (cyan, A left, B right) co-crystallized in the binding site of PPARγ and the best-ranked docking pose of **III** (blue) and their interactions with the binding site. Interactions with Ser342 and H_2_O41 are predicted.

**Fig. 6:**
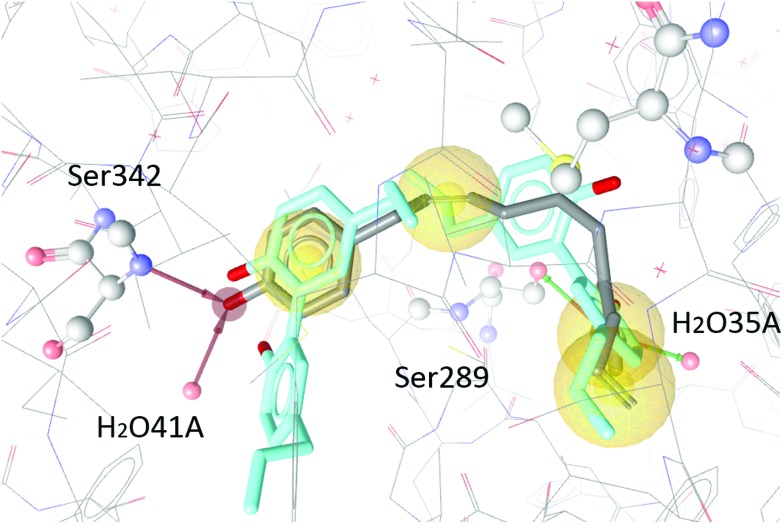
Two magnolol molecules (cyan, A left, B right) co-crystallized in the binding site of PPARγ and the best-ranked docking pose of **V** (grey) and their interactions with the binding site. Interactions with Ser342, Ser289, H_2_O41 and H_2_O35 are predicted.

The two magnolol molecules in the original crystal structure are displayed in cyan (Fig. 4–6) and referred to as magnolol A (left molecule) and magnolol B (right molecule). Magnolol A of the original crystal structure forms hydrogen bond interactions with Ser342 and a co-crystallized water molecule H_2_O41, while magnolol B binds to Ser289, Met364 and H_2_O35. Docking of sesqui magnolol B (**IV**) exhibited that the intact magnolol fragment aligned to magnolol A and formed the same interactions. The other half of the molecule filled the space occupied by magnolol B in the crystal structure of PPARγ (GoldPLP score: 110.79, Fig. 4). Sesqui magnolol A (**III**) is indeed predicted to bind in a similar pose with the intact magnolol fragment aligning with magnolol A too and forming similar interactions. (GoldPLP score: 108.21, Fig. 5) The most significant factor of the binding seems to be, whether the structure fills the available space in the binding site, especially where magnolol A binds. Both **III** and **IV** form some of the polar interactions of magnolol A and B, while **V** aligns with the two individual molecules only partly, leaving a large part of the pocket empty, albeit with considerable predicted interactions. This leads to many different proposed docking poses within the binding site and a lower score of 90.80. The program not being able to predict a stable energetic minimum for ligand binding points towards a lower affinity for the target, which corresponds with the score and the experimental findings.

**Fig. 7:**
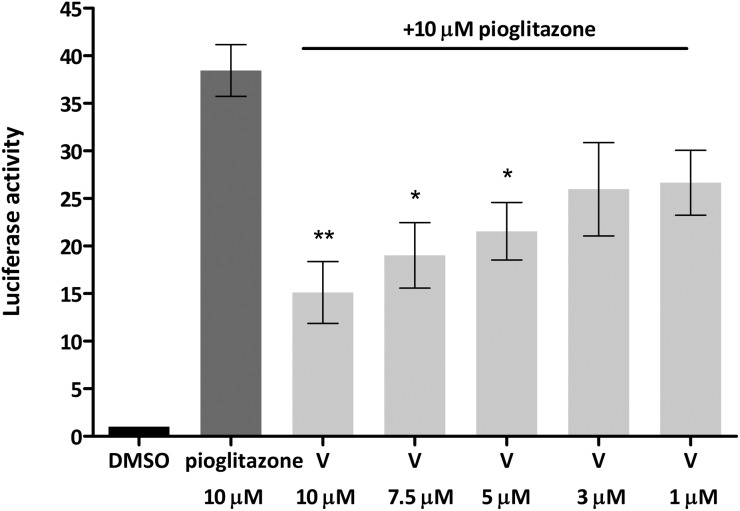
Truncated magnolol dimer (**V**) dose-dependently inhibits the pioglitazone-induced PPARγ-Gal4 mediated Luciferase gene expression. Bars show mean ± SEM. **p < 0.01, *p < 0.05 in comparison to pioglitazone, one-way ANOVA with Tukey's *post hoc* test.

Given that truncated magnolol dimer (**V**) did not show activation of the PPARγ-Gal4 receptor, but docking studies indicated that **V** can fit into the PPARγ ligand binding pocket, this suggested that **V** could interfere with the PPARγ agonist binding and thus act as an antagonist. We tested this possibility by employing the same approach by co-treating the cells with constant dose of PPARγ full agonist pioglitazone (10 μM) and **V** as indicated in Fig. 7. Indeed, **V** showed a dose-dependent inhibition of the pioglitazone induced PPARγ-Gal4 activation.

**Fig. 8:**
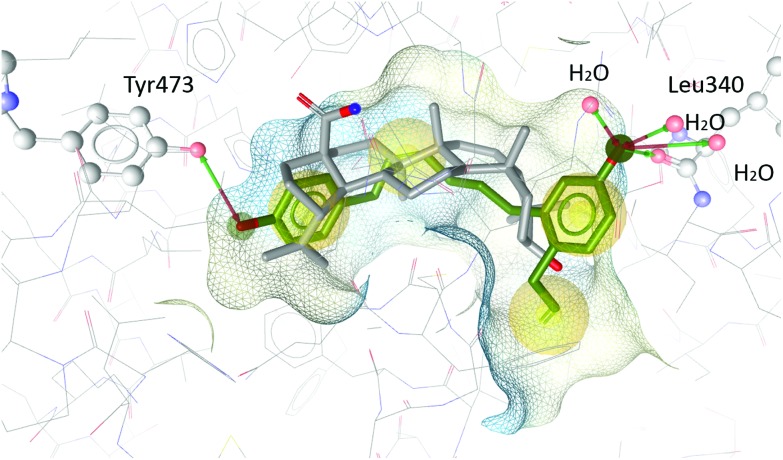
Betulinic acid (grey) co-crystallized in the binding site of PPARγ and the best-ranked docking pose of **V** (green). Interactions with Tyr473, Leu340 and three water molecules are predicted.

In order to understand the antagonistic binding behaviour of **V**, the compound was additionally docked into an antagonist structure of PPARγ, cocrystallized with betulinic acid.^24^ In this antagonist structure, **V** was docked in more conclusive poses than in the agonist structure, moving deeper into the binding pocket and forming different interactions. Compound **V** occupied a similar space in the pocket as the antagonist betulinic acid and forms interactions with Leu340 and Tyr473 and several water molecules (see Fig. 8).

## Conclusions

In this report we have designed three novel fragments of the previously disclosed magnolol dimer (**II**) structure as molecular probes for PPARγ. The new ligands have been successfully synthesized in a straight-forward fashion. In a cell-based reporter gene assay the sesqui magnolol analogs **III** and **IV** displayed very similar activity among them and compared to magnolol dimer (**II**)^14^ as well acting as partial agonists. The hypothesized common pharmacophore of **III** and **IV** was further substantiated by computational docking studies. This suggests that a single biphenyl motif in combination with another aryl domain is sufficient for receptor activation and interactions with Ser342 and H_2_O41 emerged as crucial features for activity from *in silico* methods. In contrast to the natural product magnolol (**I**), the synthetic ligands did not activate RXRα and, hence, serve as a tool for selective PPARγ activation. In truncated magnolol dimer (**V**) the biphenyl domain is not intact anymore which results in a complete loss of activation. Due to reasonable interaction predicted by docking studies, we experimentally found **V** to act as a PPARγ antagonist instead. These novel tool compounds, designed by a reversed fragment-based strategy, serve as promising selective PPARγ activators and in combination with the gained insight into ligand–receptor interactions should guide future ligand design.

## Experimental

### General methods

Chemicals were purchased from commercial suppliers and were used without further purification unless otherwise noted. For thin layer chromatography (TLC) aluminium backed silica gel 60 F254 (from Merck) was used. Flash column chromatography was performed with a Büchi Sepacore MPLC system using silica gel 60 M (particle size 40–63 μm) or conventional glass columns. Preparative TLC was performed on glass backed silica gel GF uniplates (1000 microns) from Analtech. Melting points were measured on a Büchi B-545 melting point apparatus and are uncorrected. ^1^H NMR and ^13^C NMR spectra were recorded from CDCl_3_ solutions on a Bruker AC 200 (200 MHz), a Bruker Avance UltraShield 400 (400 MHz) or a Bruker Avance III HD 600 (600 MHz) spectrometer. Chemical shifts are reported in ppm relative to the nominal residual solvent signals of CDCl_3_: ^1^H NMR: 7.26 ppm, ^13^C NMR: 77.16 ppm. HRMS data was measured with a Shimadzu HPLC-IT-TOF mass spectrometer (ESI).

#### 3-Bromo-4-methylphenol (**2**)

NaNO_2_ (2.05 g, 29.7 mmol, 1.1 equiv., dissolved in 20 mL H_2_O) was added dropwise to a suspension of 3-bromo-4-methylaniline (**1**, 5.015 g, 27.0 mmol, 1 equiv.) in diluted H_2_SO_4_ (134 mL, 40% v/v) at 0 °C and the reaction was stirred for 5 minutes Subsequently, diluted H_2_SO_4_ (35 mL H_2_O + 35 mL H_2_SO_4_) was added and the reaction was heated to 90–100 °C for 15 minutes. The reaction mixture was extracted with diethyl ether (3 × 100 mL). The combined organic layers were washed with brine (100 mL) and dried over MgSO_4_, filtered and volatiles were removed *in vacuo*. The residue was purified by flash column chromatography on silica gel (LP (light petroleum) → 5% EtOAc in LP) to afford **2** (4.185 g, 22.4 mmol, 83%) as a pale red solid. mp 55.0–55.5 °C (lit.,^25^ 55.2–55.4 °C); ^1^H NMR (200 MHz, CDCl_3_) *δ* 7.12–7.03 (m, 2H), 6.70 (dd, *J* = 8.2, 2.6 Hz, 1H), 5.06 (s, 1H), 2.32 (s, 3H) ppm; ^13^C NMR (50 MHz, CDCl_3_) *δ* 153.9, 131.3, 130.2, 124.9, 119.4, 114.6, 21.9 ppm.

#### 2-Bromo-4-methoxy-1-methylbenzene (**3**)

K_2_CO_3_ (5.97 g, 43.2 mmol, 2 equiv.) was added to a solution of **2** (4.04 g, 21.6 mmol, 1 equiv.) in acetone (110 mL). The mixture was stirred for 10 minutes. Then methyl iodide (3.99 g, 28.1 mmol, 1.3 equiv.) was added and the mixture was stirred at room temperature for 24 hours. After concentration *in vacuo* the residue was partitioned between diethyl ether (50 mL) and H_2_O (50 mL). The aqu. phase was extracted with diethyl ether (2 × 25 mL). The combined organic layers were dried over MgSO_4_, filtered and volatiles were removed *in vacuo*. Purification by flash column chromatography on silica gel (LP → 2% EtOAc in LP) provided **3** (3.54 g, 17.6 mmol, 81%) as a colorless liquid. ^1^H NMR (200 MHz, CDCl_3_) *δ* 7.16–7.07 (m, 2H), 6.76 (dd, *J* = 8.4, 2.7 Hz, 1H), 3.77 (s, 3H), 2.32 (s, 3H) ppm; ^13^C NMR (50 MHz, CDCl_3_) *δ* 158.3, 131.1, 129.8, 125.0, 117.7, 113.5, 55.7, 22.0 ppm.

#### 2-Bromo-1-(bromomethyl)-4-methoxybenzene (**4**)

NBS (1.05 g, 5.89 mmol, 1 equiv.) and benzoyl peroxide (17 mg, 0.07 mmol, 1.2 mol%) were added to a solution of **3** (1.18 g, 5.89 mmol, 1 equiv.) in dry and degassed CCl_4_ (74 mL) under argon atmosphere and the reaction was stirred at reflux temperature for 2 hours. The reaction mixture was washed successively with 2 N HCl (74 mL), satd. aqu. NaHCO_3_ (74 mL), H_2_O (74 mL), and brine (74 mL). The organic phase was dried over Na_2_SO_4_, filtered and volatiles were removed *in vacuo*. Purification by flash column chromatography on silica gel (2% EtOAc in LP) provided **4** (1.42 g, 5.06 mmol, 85%) as a colorless solid. Mp 59.0–60.5 °C (lit.:^26^ 59–60 °C); ^1^H NMR (200 MHz, CDCl_3_) *δ* 7.36 (d, *J* = 8.5 Hz, 1H), 7.12 (d, *J* = 2.6 Hz, 1H), 6.84 (dd, *J* = 8.5, 2.6 Hz, 1H), 4.60 (s, 2H), 3.80 (s, 3H) ppm; ^13^C NMR (50 MHz, CDCl_3_) *δ* 160.4, 132.0, 129.2, 125.2, 118.6, 114.2, 55.8, 33.9 ppm.

#### 2-Bromo-1-iodo-4-methoxybenzene (**6**)

A mixture of 1-bromo-3-methoxybenzene (**5**, 642 mg, 3.43 mmol, 1 equiv.), HgO (744 mg, 3.43 mmol, 1 equiv.) and Ac_2_O (0.13 mL, 1.37 mmol, 0.4 equiv.) in DCM (12.7 mL) was heated to reflux temperature. Freshly sublimed I_2_ (1220 mg, 4.81 mmol, 1.4 equiv.) was added and the reaction was stirred at reflux temperature for 19 hours. Due to incomplete reaction progress, more I_2_ (523 mg, 2.06 mmol, 0.6 equiv.) was added and the reaction was stirred for another 15 hours (34 h in total) at reflux temperature. The reaction mixture was filtered through a pad of Celite and the pad was washed with DCM (in total 30 mL) and satd. aqu. Na_2_S_2_O_3_ (10 mL). Phases were separated and the aqu. phase was extracted with DCM (3 × 10 mL). The combined organic layers were washed with brine (30 mL), dried over MgSO_4_, filtered and volatiles were removed *in vacuo*. After purification by flash column chromatography on silica gel (cyclohexane) **6** (721 mg, 2.30 mmol, 67%) was obtained as a colorless liquid. ^1^H NMR (200 MHz, CDCl_3_) *δ* 7.69 (d, *J* = 8.8 Hz, 1H), 7.19 (d, *J* = 2.9 Hz, 1H), 6.60 (dd, *J* = 8.8, 2.9 Hz, 1H), 3.78 (s, 3H) ppm; ^13^C NMR (50 MHz, CDCl_3_) *δ* 160.3, 140.4, 130.1, 118.5, 115.5, 89.6, 55.7 ppm.

#### 1-Allyl-2-bromo-4-methoxybenzene (**7**)

Method A: A solution of **6** (944 mg, 3.02 mmol 1 equiv.), allyltributylstannane (1049 mg, 3.17 mmol, 1.05 equiv.) and Pd(PPh_3_)_4_ (314 mg, 0.27 mmol, 9 mol%) in dry DMF (9.5 mL) under argon was heated to 100 °C for 45 minutes. H_2_O (45 mL) was added and the mixture was extracted with EtOAc (3 × 30 mL). The combined organic layers were washed with aqu. KF (10%, 10 mL), which caused precipitation. The mixture was filtered and the organic phase was washed with aqu. KF (10%, 2 × 10 mL), brine (10 mL) and was dried over MgSO_4_, filtered and volatiles were removed *in vacuo*. Purification by flash column chromatography on silica gel (LP → 2% EtOAc in LP) afforded **7** (555 mg, 2.44 mmol, 81%) as a colorless liquid. Method B: A solution of **4** (2994 mg, 10.7 mmol, 1 equiv.) in THF was added to a mixture of CuI (204 mg, 1.07 mmol, 10 mol%), 2,2′-bipyridyl (167 mg, 1.07 mmol, 10 mol%) and vinylmagnesium bromide(21.4 mL, 21.4 mmol, 2 equiv., 1 M in THF) under argon at 5 °C. The mixture was stirred for one hour at that temperature whereupon the reaction was quenched with satd. aqu. NH_4_Cl (60 mL). Diethyl ether (60 mL) and conc. ammonia (4.5 mL) were added and phases were separated. The aqu. layer was extracted with diethyl ether (2 × 30 mL). The combined organic layers were washed with 2 N HCl (75 mL) and satd. aqu. NaHCO_3_ (75 mL), dried over MgSO_4_, filtered and volatiles were removed *in vacuo*. After purification by flash column chromatography on silica gel (LP → 2% EtOAc in LP) **7** was isolated as a 3/1 mixture contaminated with its toluene analog in a calculated yield of 36% (874 mg, 3.85 mmol). ^1^H NMR (200 MHz, CDCl_3_) *δ* 7.16–7.07 (m, 2H), 6.81 (dd, *J* = 8.5, 2.6 Hz, 1H), 5.95 (ddt, *J* = 16.7, 10.3, 6.4 Hz, 1H), 5.15–4.97 (m, 2H), 3.78 (s, 3H), 3.44 (dt, *J* = 6.5, 1.6 Hz, 2H) ppm; ^13^C NMR (50 MHz, CDCl_3_) *δ* 158.7, 136.2, 131.5, 130.9, 124.7, 118.0, 116.3, 113.8, 55.7, 39.4 ppm; HRMS (ESI) calcd for C_10_H_12_BrO^+^ (M + H)^+^ 227.0066, found 227.0061.

#### 1-Allyl-2-(4-chlorobutyl)-4-methoxybenzene (**8**)

*t*-BuLi (4.12 mL, 7.00 mmol, 2 equiv., 1.7 M in pentane) was added dropwise to a solution of **7** (795 mg, 3.50 mmol, 1 equiv.) in dry THF (7 mL) under argon at –78 °C and the reaction was stirred for 15 minutes at –78 °C and 10 minutes at –50 °C. The reaction was cooled to –78 °C and 1-bromo-4-chlorobutane (900 mg, 5.25 mmol, 1.5 equiv.) was added whereupon the reaction was warmed to room temperature to be stirred subsequently for 1 hour. The reaction solution was then partitioned between satd. aqu. NH_4_Cl (30 mL) and DCM (30 mL). The aqu. layer was extracted with DCM (2 × 10 mL) and the combined organic layers were dried over MgSO_4_, filtered and volatiles were removed *in vacuo*. After purification by flash column chromatography on silica gel (5% → 15% DCM in LP) **8** (654 mg, 2.74 mmol, 78%) was obtained as a colorless liquid. ^1^H NMR (200 MHz, CDCl_3_) *δ* 7.07 (d, *J* = 9.2 Hz, 1H), 6.75–6.66 (m, 2H), 5.95 (ddt, *J* = 16.5, 10.2, 6.2 Hz, 1H), 5.09–4.90 (m, 2H), 3.79 (s, 3H), 3.56 (t, *J* = 6.3 Hz, 2H), 3.34 (dt, *J* = 6.2, 1.7 Hz, 2H), 2.67–2.55 (m, 2H), 1.94–1.64 (m, 4H) ppm; ^13^C NMR (50 MHz, CDCl_3_) *δ* 158.2, 141.4, 137.8, 130.8, 129.8, 115.5, 115.1, 111.3, 55.3, 45.0, 36.4, 32.6, 32.2, 28.1 ppm; HRMS (ESI) calcd for C_14_H_20_ClO^+^ (M + H)^+^ 239.1197, found 239.1193.

#### (4-(2-Allyl-5-methoxyphenyl)butyl)triphenylphosphonium iodide (**9**)

A mixture of **8** (113 mg, 0.47 mmol, 1 equiv.), NaI (71 mg, 0.47 mmol, 1 equiv.) and PPh_3_ (124 mg, 0.47 mmol, 1 equiv.) in EtOAc (2.37 mL) under argon was stirred at reflux temperature for 65 hours. Volatiles were removed *in vacuo*. CHCl_3_ was added and after stirring for 5 minutes the mixture was filtered through a pad of Na_2_SO_4_. Volatiles were removed *in vacuo* and after drying in high vacuum, the crude product was obtained as an oil. Stirring with refluxing diethyl ether prompted the oil to crystallize. After cooling to room temperature the solvent was decanted and after drying in high vacuum **9** (210 mg, 0.35 mmol, 75%) was obtained as beige crystals. Mp 121.0–124.0 °C; ^1^H NMR (400 MHz, CDCl_3_) *δ* 7.81–7.75 (m, 9H), 7.71–7.65 (m, 6H), 6.98 (d, *J* = 8.3 Hz, 1H), 6.69 (d, *J* = 2.7 Hz, 1H), 6.65 (dd, *J* = 8.3, 2.7 Hz, 1H), 5.84 (ddt, *J* = 17.2, 10.1, 6.2 Hz, 1H), 4.95–4.82 (m, 2H), 3.78–3.67 (m, 5H), 3.22 (dt, *J* = 6.3, 1.7 Hz, 2H), 2.60 (t, *J* = 7.5 Hz, 2H), 1.96 (quin, *J* = 7.5 Hz, 2H), 1.73–1.62 (m, 2H) ppm; ^13^C NMR (101 MHz, CDCl_3_) *δ* 158.2, 140.6, 137.9, 135.2 (d, ^4^*J*_CP_ = 3.0 Hz, 3C), 133.8 (d, ^3^*J*_CP_ = 10.0 Hz, 6C), 130.7, 130.6 (d, ^2^*J*_CP_ = 12.4 Hz, 6C), 129.6, 118.3 (d, ^1^*J*_CP_ = 85.9 Hz, 3C), 115.4, 114.7, 111.9, 55.7, 36.4, 32.1, 31.0 (d, ^2^*J*_CP_ = 15.4 Hz), 23.2 (d, ^1^*J*_CP_ = 49.9 Hz), 22.3 (d, ^3^*J*_CP_ = 4.4 Hz) ppm; HRMS (ESI) calcd for C_32_H_34_OP^+^ (M – I)^+^ 465.2342, found 465.2352.

#### (4-(5,5′-Diallyl-2,2′-dimethoxy-[1,1′-biphenyl]-4-yl)butyl)triphenylphosphonium iodide (**10**)

See ref. 14.

#### 2-(4-Methoxyphenyl)acetaldehyde (**11**)

See ref 14.

#### 2-(5′-Allyl-2′,6-dimethoxy-[1,1′-biphenyl]-3-yl)acetaldehyde (**12**)

See ref. 14.

#### (*Z*)-1-Allyl-4-methoxy-2-(6-(4-methoxyphenyl)hex-4-en-1-yl)benzene (**13**)

KHMDS (4.05 mL, 2.03 mmol, 1.5 equiv., 0.5 M in toluene) was added to a suspension of Wittig reagent **9** (800 mg, 1.35 mmol, 1 equiv.) in dry diethyl ether (6.75 mL) under argon at 0 °C. The reaction was stirred for 5 minutes at 0 °C and 20 minutes at room temperature. Afterwards, the reaction was cooled to –55 °C and aldehyde **11** (304 mg, 2.03 mmol, 1.5 equiv.) was added as a solution in dry diethyl ether. The reaction was allowed to warm up and was stirred at room temperature for 5 hours. The mixture was dried over MgSO_4_, filtered and volatiles were removed *in vacuo*. Purification by flash column chromatography on silica gel (1% → 5% EtOAc in LP) afforded **13** (386 mg, 1.15 mmol, 85%) as a colorless oil. ^1^H NMR (200 MHz, CDCl_3_) *δ* 7.15–7.02 (m, 3H), 6.88–6.79 (m, 2H), 6.76–6.67 (m, 2H), 5.95 (ddt, *J* = 16.6, 10.2, 6.3 Hz, 1H), 5.69–5.44 (m, 2H), 5.09–4.91 (m, 2H), 3.79 (s, 6H), 3.40–3.28 (m, 4H), 2.68–2.56 (m, 2H), 2.24 (q, *J* = 7.0 Hz, 2H), 1.69 (p, *J* = 7.6 Hz, 2H) ppm; ^13^C NMR (50 MHz, CDCl_3_) *δ* 158.2, 157.9, 142.0, 137.9, 133.2, 130.6, 130.1, 129.8, 129.3 (2C), 129.2, 115.4, 115.0, 114.0 (2C), 111.2, 55.4, 55.3, 36.4, 32.7, 30.9, 27.3 ppm; HRMS (ESI) calcd for C_23_H_29_O_2_^+^ (M + H)^+^ 337.2162, found 337.2163.

#### (*Z*)-5,5′-Diallyl-2,2′-dimethoxy-4-(6-(4-methoxyphenyl)hex-4-en-1-yl)-1,1′-biphenyl (**14**)

Three identical batches were set up. KHMDS (122 μL, 0.06 mmol, 1.5 equiv., 0.5 M in toluene) was added to a suspension of Wittig reagent **10** (30 mg, 0.04 mmol, 1 equiv.) in dry diethyl ether (1 mL) under argon at 0 °C. The reaction was stirred for 5 minutes at 0 °C and 20 minutes at room temperature. Afterwards, the reaction was cooled to –55 °C and aldehyde **11** (9 mg, 0.06 mmol, 1.5 equiv.) was added as a solution in dry diethyl ether. The reaction was allowed to warm up and was stirred at room temperature for 5 hours. The three identical batches were combined and the mixture was dried over MgSO_4_, filtered and volatiles were removed *in vacuo*. Purification by flash column chromatography on silica gel (1% → 5% EtOAc in LP) afforded **14** (48 mg, 0.10 mmol, 82%) as a colorless oil. ^1^H NMR (400 MHz, CDCl_3_) *δ* 7.16–7.10 (m, 3H), 7.07 (d, *J* = 2.3 Hz, 1H), 7.03 (s, 1H), 6.90 (d, *J* = 8.4 Hz, 1H), 6.84 (d, *J* = 8.6 Hz, 2H), 6.78 (s, 1H), 6.05–5.90 (m, 2H), 5.67–5.53 (m, 2H), 5.14–5.00 (m, 4H), 3.79 (s, 3H), 3.76 (s, 6H), 3.44–3.33 (m, 6H), 2.71–2.64 (m, 2H), 2.29 (q, *J* = 7.1 Hz, 2H), 1.75 (p, *J* = 7.6 Hz, 2H) ppm; ^13^C NMR (101 MHz, CDCl_3_) *δ* 158.0, 155.7, 155.6, 140.9, 138.0, 137.9, 133.3, 132.7, 131.9, 131.9, 130.2, 129.5, 129.3 (2C), 129.2, 128.4, 127.9, 125.6, 115.6, 115.5, 114.0 (2C), 112.3, 111.3, 56.0, 56.0, 55.4, 39.6, 36.6, 32.9, 32.8, 31.1, 27.5 ppm; HRMS (ESI) calcd for C_33_H_39_O_3_^+^ (M + H)^+^ 483.2894, found 483.2870.

#### (*Z*)-5-Allyl-5′-(6-(2-allyl-5-methoxyphenyl)hex-2-en-1-yl)-2,2′-dimethoxy-1,1′-biphenyl (**15**)

KHMDS (0.61 mL, 0.31 mmol, 1.5 equiv., 0.5 M in toluene) was added to a suspension of Wittig reagent **9** (121 mg, 0.20 mmol, 1 equiv.) in dry diethyl ether (1.02 mL) under argon at 0 °C. The reaction was stirred for 5 minutes at 0 °C and 20 minutes at room temperature. Afterwards, the reaction was cooled to –55 °C and aldehyde **12** (91 mg, 0.31 mmol, 1.5 equiv.) was added as a solution in dry diethyl ether. The reaction was allowed to warm up and was stirred at room temperature for 5 hours. The mixture was dried over MgSO_4_, filtered and volatiles were removed *in vacuo*. Purification by flash column chromatography on silica gel (1% → 5% EtOAc in LP) afforded **15** (79 mg, 0.16 mmol, 80%) as a colorless oil. ^1^H NMR (400 MHz, CDCl_3_) *δ* 7.14–7.10 (m, 2H), 7.07–7.03 (m, 3H), 6.89 (dd, *J* = 8.4, 2.0 Hz, 2H), 6.73 (d, *J* = 2.7 Hz, 1H), 6.70 (dd, *J* = 8.2, 2.8 Hz, 1H), 6.04–5.87 (m, 2H), 5.67–5.49 (m, 2H), 5.12–4.93 (m, 4H), 3.77 (s, 3H), 3.75 (s, 3H), 3.74 (s, 3H), 3.40–3.29 (m, 6H), 2.63–2.57 (m, 2H), 2.23 (dt, *J* = 7.9, 6.4 Hz, 2H), 1.72–1.63 (m, 2H) ppm; ^13^C NMR (101 MHz, CDCl_3_) *δ* 158.2, 155.6, 155.5, 142.0, 137.9, 137.9, 132.8, 131.9, 131.7, 131.5, 130.6, 130.1, 129.8, 129.2, 128.5, 128.3, 128.0, 115.6, 115.4, 115.0, 111.3, 111.2, 111.2, 56.0, 56.0, 55.3, 39.5, 36.4, 32.8, 32.7, 31.0, 27.3 ppm; HRMS (ESI) calcd for C_33_H_39_O_3_^+^ (M + H)^+^ 483.2894, found 483.2906.

#### Truncated magnolol dimer (**V**)

A mixture of **13** (50 mg, 0.15 mmol, 1 equiv.) and BBr_3_·S(CH_3_)_2_ (112 mg, 0.36 mmol, 2.4 equiv.) in dry 1,2-dichloroethane (1 mL) under argon was stirred at reflux temperature for 2 hours. H_2_O (1 mL) was added and the two phases were separated. The aqu. phase was extracted with 1,2-dichloroethane (2 × 1 mL) and the combined organic layers were washed with brine (2 mL), dried over MgSO_4_, filtered and volatiles were removed *in vacuo*. After purification by preparative TLC on silica gel (DCM/MeOH = 9/1) **V** (30 mg, 0.10 mmol, 66%) was obtained as a colorless oil. ^1^H NMR (600 MHz, CDCl_3_) *δ* 7.05 (d, *J* = 8.4 Hz, 2H), 7.02–6.99 (m, 1H), 6.77 (d, *J* = 8.5 Hz, 2H), 6.65–6.62 (m, 2H), 5.94 (ddt, *J* = 16.6, 10.1, 6.3 Hz, 1H), 5.62–5.50 (m, 2H), 5.11 (s, 1H), 5.06–4.96 (m, 3H), 3.34–3.30 (m, 4H), 2.59–2.55 (m, 2H), 2.20 (q, *J* = 7.3 Hz, 2H), 1.65 (p, *J* = 7.7 Hz, 2H) ppm; ^13^C NMR (151 MHz, CDCl_3_) *δ* 153.9, 153.7, 142.2, 137.8, 133.5, 130.9, 130.3, 130.0, 129.5 (2C), 129.1, 116.1, 115.5, 115.4 (2C), 113.0, 36.4, 32.7, 32.4, 30.7, 27.2 ppm; HRMS (ESI) calcd for C_21_H_23_O_2_^–^ (M – H)^–^ 307.1704, found 307.1693.

#### Sesqui magnolol A (**III**)

A mixture of **14** (30 mg, 0.06 mmol, 1 equiv.) and BBr_3_·S(CH_3_)_2_ (68 mg, 0.22 mmol, 3.5 equiv.) in dry 1,2-dichloroethane (1.5 mL) under argon was stirred at reflux temperature for 21 hours. H_2_O (2 mL) was added and the two phases were separated. The aqu. layer was extracted with 1,2-dichloroethane (3 × 2 mL) and the combined organic layers were washed with brine (4 mL), dried over MgSO_4_, filtered and volatiles were removed *in vacuo*. After purification by flash column chromatography on silica gel (1% MeOH in CH_3_Cl) **III** (17 mg, 0.04 mmol, 62%) was obtained as a colorless oil. ^1^H NMR (400 MHz, CDCl_3_) *δ* 7.12 (dd, *J* = 8.2, 2.3 Hz, 1H), 7.09–7.02 (m, 4H), 6.96 (d, *J* = 8.3 Hz, 1H), 6.82 (s, 1H), 6.76 (d, *J* = 8.4 Hz, 2H), 5.95 (dddt, *J* = 16.5, 10.1, 7.5, 6.5 Hz, 2H), 5.69–5.44 (m, 4H), 5.13–4.98 (m, 4H), 4.83 (s, 1H), 3.39–3.32 (m, 6H), 2.65–2.58 (m, 2H), 2.24 (q, *J* = 7.1 Hz, 2H), 1.74–1.65 (m, 2H) ppm; ^13^C NMR (101 MHz, CDCl_3_) *δ* 153.8, 151.4, 151.2, 142.8, 137.7, 137.5, 133.5, 133.2, 132.3, 131.3, 131.0, 130.3, 129.9, 129.6 (2C), 129.2, 123.8, 121.4, 117.2, 116.8, 115.9, 115.8, 115.5 (2C), 39.5, 36.5, 32.8, 32.3, 30.7, 27.3 ppm; HRMS (ESI) calcd for C_30_H_31_O_3_^–^ (M – H)^–^ 439.2279, found 439.2297.

#### Sesqui magnolol B (**IV**)

A mixture of **15** (40 mg, 0.08 mmol, 1 equiv.) and BBr_3_·S(CH_3_)_2_ (91 mg, 0.29 mmol, 3.5 equiv.) in dry 1,2-dichloroethane (2 mL) under argon was stirred at reflux temperature for 21 hours. H_2_O (2 mL) was added and the two layers were separated. The aqu. layer was extracted with 1,2-dichloroethane (3 × 2 mL) and the combined organic layers were washed with brine (4 mL), dried over MgSO_4_, filtered and volatiles were removed *in vacuo*. After purification by flash column chromatography on silica gel (1% MeOH in CH_3_Cl) **IV** (23 mg, 0.05 mmol, 63%) was obtained as a colorless oil. ^1^H NMR (400 MHz, CDCl_3_) *δ* 7.13 (dt, *J* = 8.2, 2.0 Hz, 2H), 7.08 (t, *J* = 2.7 Hz, 2H), 7.00–6.93 (m, 3H), 6.63–6.57 (m, 2H), 6.02–5.85 (m, 2H), 5.68–5.50 (m, 4H), 5.12–4.92 (m, 4H), 4.78 (s, 1H), 3.41–3.26 (m, 6H), 2.59–2.51 (m, 2H), 2.24–2.16 (m, 2H), 1.69–1.59 (m, 2H) ppm; ^13^C NMR (101 MHz, CDCl_3_) *δ* 154.0, 151.3, 151.2, 142.1, 137.9, 137.6, 134.4, 133.4, 131.3, 131.0, 130.9, 130.7, 130.2, 129.9, 129.9, 128.7, 123.8, 123.8, 116.9, 116.8, 116.0, 116.0, 115.5, 113.0, 39.5, 36.4, 32.7, 32.4, 30.8, 27.2 ppm; HRMS (ESI) calcd for C_30_H_33_O_3_^+^ (M + H)^+^ 441.2424, found 441.2436.

#### Magnolol dimer (**II**)

See ref. 14.

HEK293T cells were purchased from ATCC (Manassas, USA), Dulbeccós Modified Eagle Medium (DMEM; 4.5 g L^–1^ glucose) from Lonza (Basel; Switzerland) and Fetal Bovine Serum (FBS) from ThermoFisher Scientific (Waltham, USA). Plasmids pCMX-Gal4-hPPARγ, pCMX-Gal4-hRXRα, tk(MH1000)-4xLuc and tk-PPREx3-Luc were a gift from Dr Ronald Evans (Salk Institute, La Jolla, USA). Plasmids pSG5-hPPARγ1 and pSG5-hRXRα were a gift from Prof. Walter Wahli and Prof. Beatrice Desvergne (Center for Integrative Genomics, University of Lausanne, Switzerland). pEGFP-N1 was purchased from Takara Bio USA (Mountain View, USA). Magnolol was isolated as described in the literature.^9^ Pioglitazone was purchased from Molekula (Munich, Germany), 9-*cis*-retinoic acid from Cayman Chemical (Ann Arbor, USA). All compounds are stored as solutions dissolved in DMSO.

### Biological evaluation methods

Luciferase reporter assays were performed in the HEK293 cell line. Cells were cultivated under standard conditions (37 °C, 5% CO_2_, passage every 3 days) in DMEM Complete containing 10% FBS, 2 mM glutamine, 100 U mL^–1^ benzylpenicillin and 100 μg mL^–1^ streptomycin. For transfections, 6 × 10^6^ cells were seeded in 15 cm Petri dishes and incubated overnight. Cells were then transfected using the calcium phosphate method with the following plasmids: for PPARγ-Gal4 and RXRα-Gal4 experiments: 6 μg of pCMX-Gal4-hPPARγ or pCMX-Gal4-hRXRα, respectively and 6 μg tk(MH1000)-4xLuc reporter plasmid were used. Additionally, all cells were co-transfected with 3 μg of pEGFP-N1 to control for transfection efficiency. After 6 hours, cells were washed with PBS and medium was replaced with 5% charcoal-stripped serum DMEM (supplemented with glutamine and antibiotics as DMEM Complete). For compound treatments, 3 × 10^5^ cells per well were seeded in a 96-well plate. Cells were then treated with 0.1% DMSO as vehicle control or with appropriate dilutions of compounds and incubated for 18 hours. Cells were lysed using the Promega Luciferase Cell Culture Lysis 5X Reagent (Mannheim, Germany). Luminescence and fluorescence values were measured on a Tecan Spark instrument (Männedorf, Switzerland). Luminescence values were normalized to EGFP fluorescence and expressed as fold changes relative to the DMSO control. All experiments were performed minimum three times, each with four technical replicates. Data was analyzed using the GraphPad Prism 4 Software (La Jolla).

### Docking

The ligands were constructed using ChemDraw 15 Professional (PerkinElmer Inc.) and a Pipeline Pilot 8 (Dassault Systems Inc.) protocol translating .cdx into .sd files. The docking simulations were carried out in Gold 5.2 (CCDC, www.ccdc.cam.ac.uk, 2015; Cambridge Crystallographic Data Centre, UK; ; www.ccdc.cam.ac.uk/solutions/csd-discovery/components/gold/).^27^,^28^ Docking poses and protein–ligand interactions were visualized in LigandScout 4.1 (Inte:Ligand, ; http://www.inteligand.com/, 2017).

For docking into the PPARγ receptor, the X-ray crystal structure of PPARγ 3R5N, a structure of human PPARγ co-crystalized with a magnolol dimer, was employed.^10^ ChemScore kinase was selected as a configuration template and CHEMPLP was selected as a scoring function.

The protonation state of His323 was set to NE2 and the water molecule no. 35 was set to “toggle and spin”. This means that the docking algorithm can choose to turn, keep or delete this water molecule depending on which setting gives the best docking results. For the docking, both magnolol ligands were removed from the binding site and used to define the binding site location. Docking settings were validated by re-docking of magnolol into the receptor. Magnolol was docked either at the location of one or the other co-crystallized magnolol binding sites with an average RMSD of 0.823 Å.

For docking into the antagonist PPARγ structure, the X-ray crystal structure of PPARγ 5LSG, a structure of human PPARγ co-crystalized with betulinic acid, was employed.^24^

## Conflicts of interest

There are no conflicts to declare.

## Supplementary Material

Supplementary information
