# Supplementary material for: Magnolol dimer-derived fragments as PPARγ-selective probes
Source: Org Biomol Chem. 2018 Sep 20;16(38):7019–28. doi: 10.1039/c8ob01745j (PMC6180429; doi:10.1039/c8ob01745j)

Electronic Supplementary Material (ESI) for Organic & Biomolecular Chemistry.
This journal is © The Royal Society of Chemistry 2018

Electronic Supplementary Information (ESI) for

Magnolol dimer-derived fragments as PPARγ-selective probes

Dominik Dreier,[a] Mirta Resetar,[b] Veronika Temml,[c] Lukas Rycek,[a] Nicolas Kratena,[a] Michael Schnürch,[a] Daniela Schuster,[d] Verena M. Dirsch[b] and Marko D. Mihovilovic*[a]

[a] *Institute of Applied Synthetic Chemistry, TU Wien, Getreidemarkt 9/163, A-1060 Vienna, Austria. E-mail: marko.mihovilovic@tuwien.ac.at*
[b] *Department of Pharmacognosy, University of Vienna, Althanstraße 14, 1090 Vienna, Austria*
[c] *Institute of Pharmacy/Pharmacognosy and Center for Molecular Biosciences Innsbruck, University of Innsbruck, Innrain 80/82, 6020 Innsbruck, Austria*
[d] *Institute of Pharmacy/Pharmaceutical Chemistry and Center for Molecular Biosciences Innsbruck, University of Innsbruck, Innrain 80/82, 6020 Innsbruck, Austria; Paracelsus Medical University Salzburg, Institute of Pharmacy, Department of Pharmaceutical and Medicinal Chemistry, Strubergasse 21, 5020 Salzburg, Austria.*

Table of contents

NMR spectra of **2** 2
NMR spectra of **3** 3
NMR spectra of **4** 4
NMR spectra of **6** 5
NMR spectra of **7** 6
NMR spectra of **8** 7
NMR spectra of **9** 8
NMR spectra of **13** 9
NMR spectra of **14** 10
NMR spectra of **15** 11
NMR spectra of **III** 12
NMR spectra of **IV** 13
NMR spectra of **V** 14

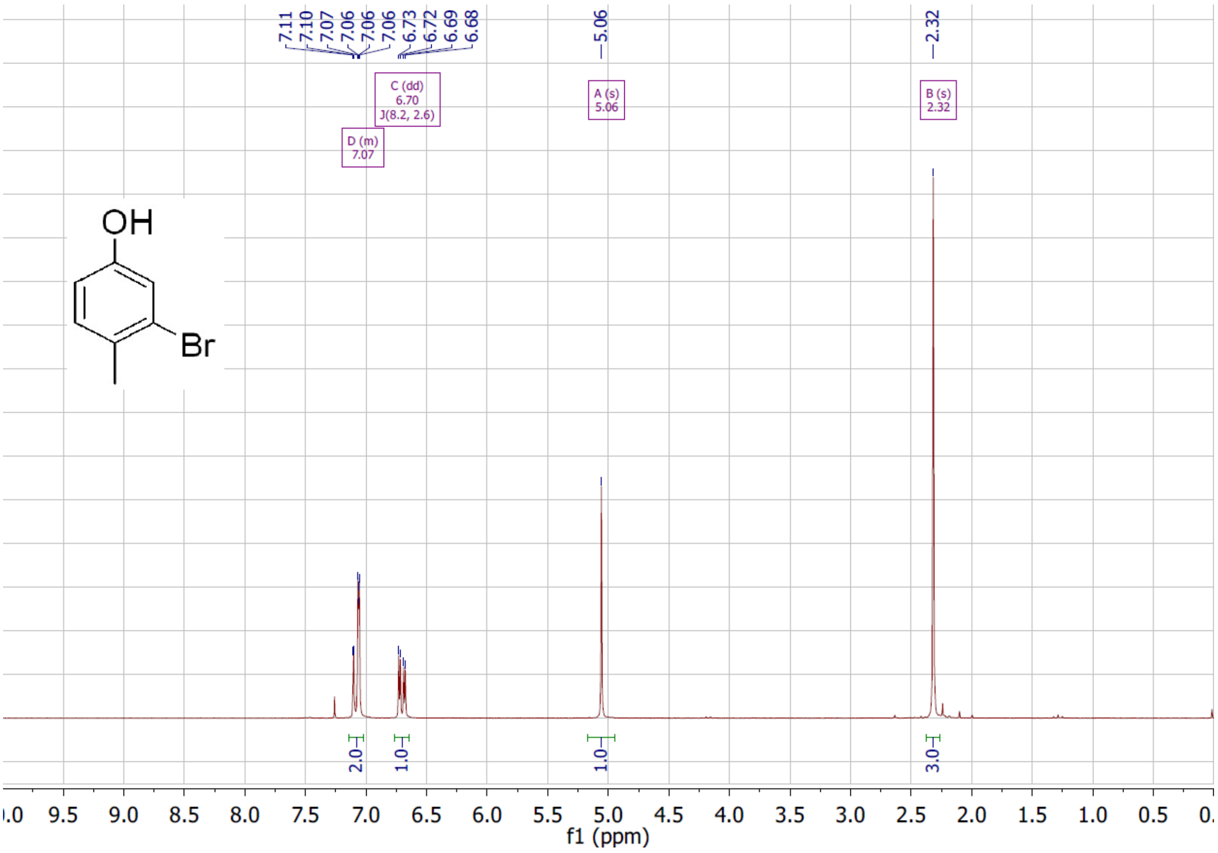

7.11
7.10
7.07
7.06
7.06
7.06
6.73
6.72
6.69
6.68
5.06
2.32
C (dd)
6.70
J(8.2, 2.6)
D (m)
7.07
A (s)
5.06
B (s)
2.32
OH
Br
2.0
1.0
1.0
3.0
f1 (ppm)

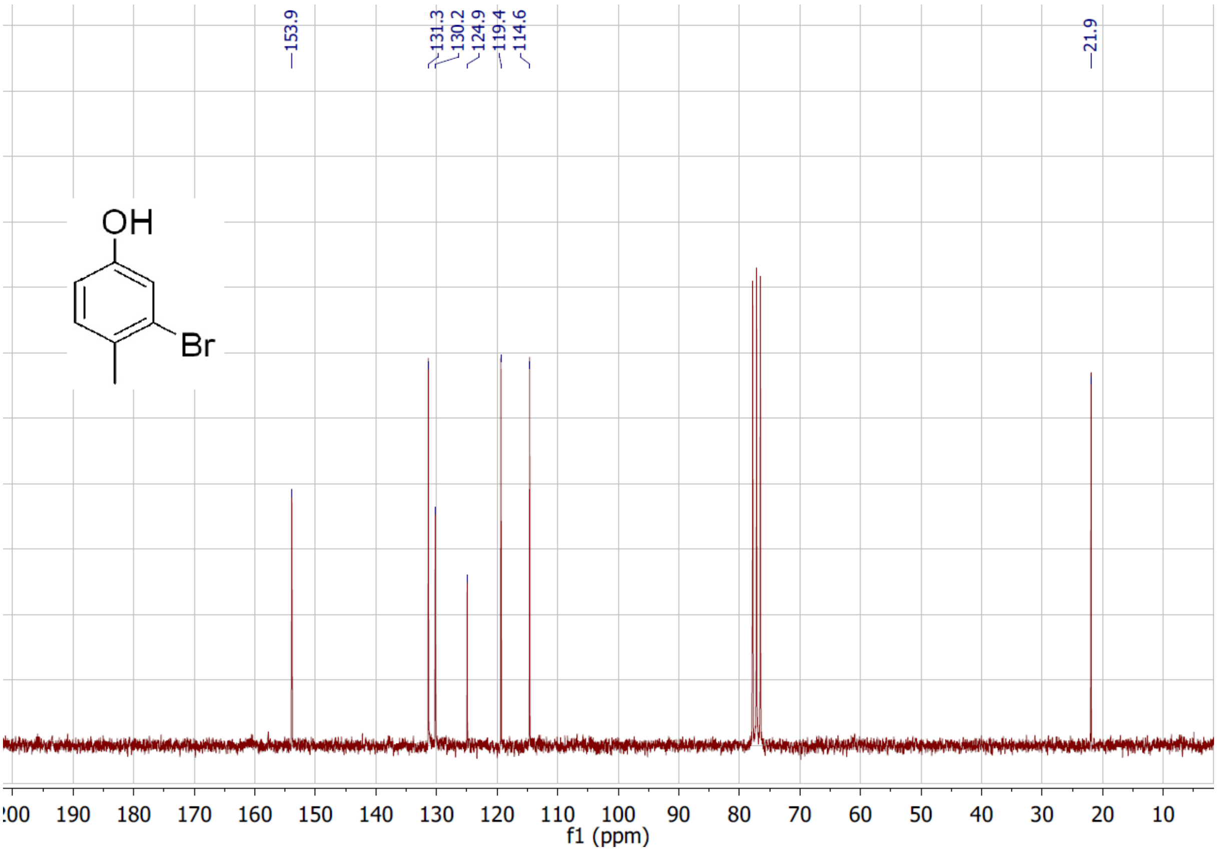

153.9
131.3
130.2
124.9
119.4
114.6
21.9
OH
Br
f1 (ppm)

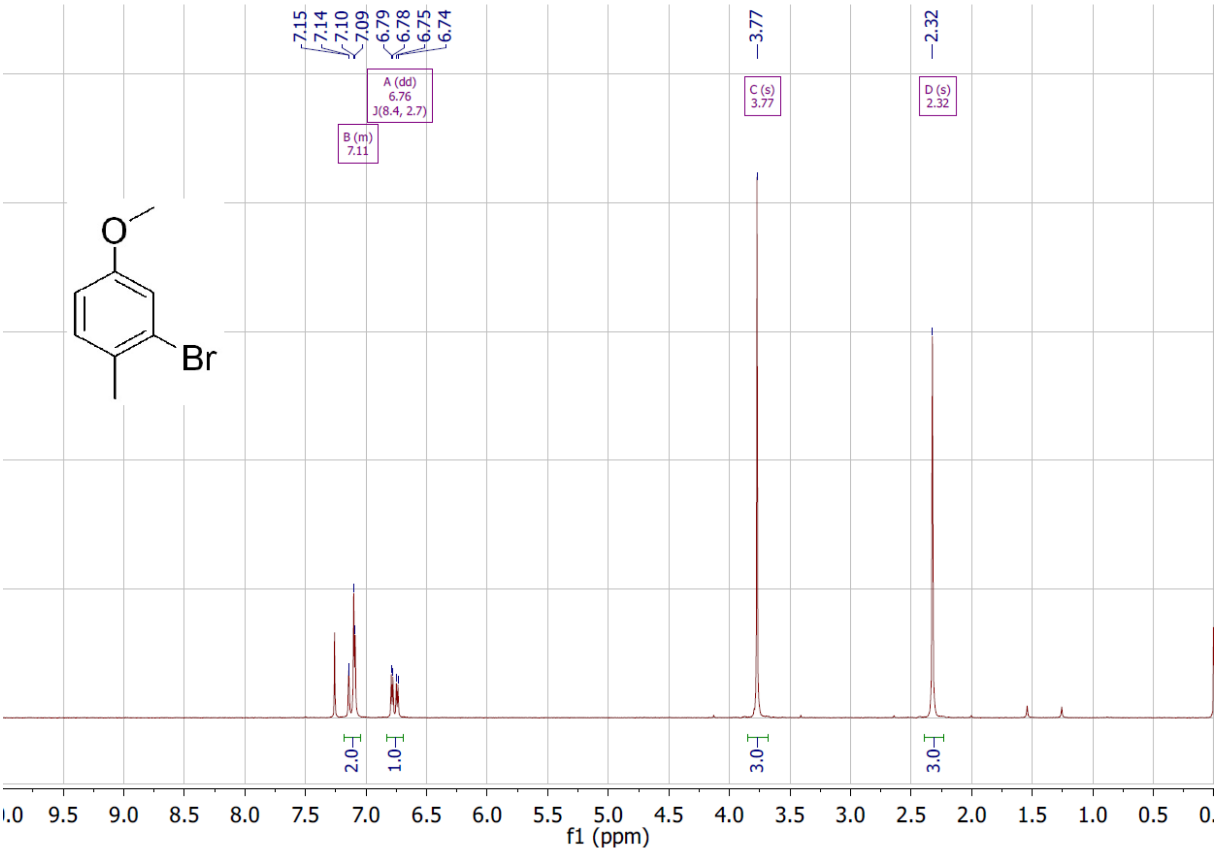
7.15
7.14
7.10
7.09
6.79
6.78
6.75
6.74
3.77
2.32
A (dd) 6.76 J(8.4, 2.7)
B (m) 7.11
C (s) 3.77
D (s) 2.32
O
Br
2.0
1.0
3.0
3.0
f1 (ppm)

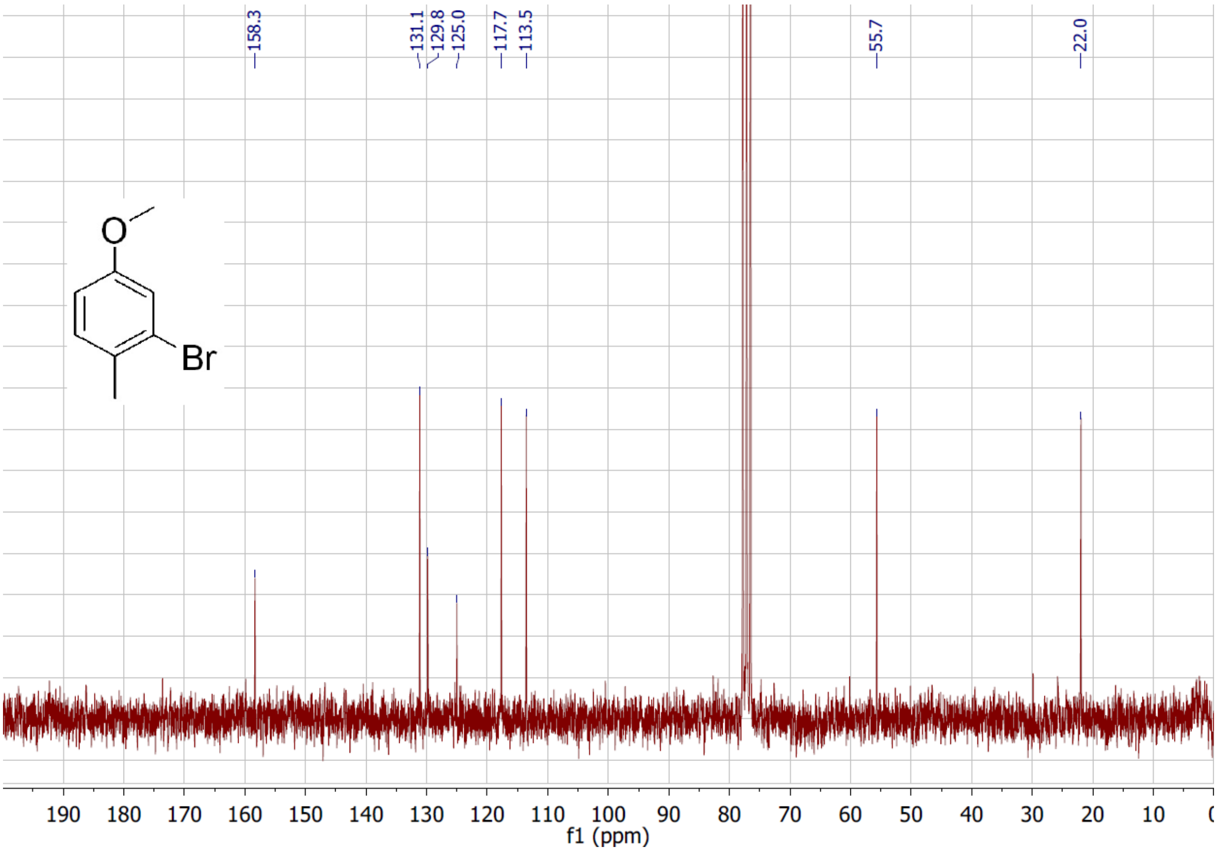
158.3
131.1
129.8
125.0
117.7
113.5
55.7
22.0
O
Br
f1 (ppm)

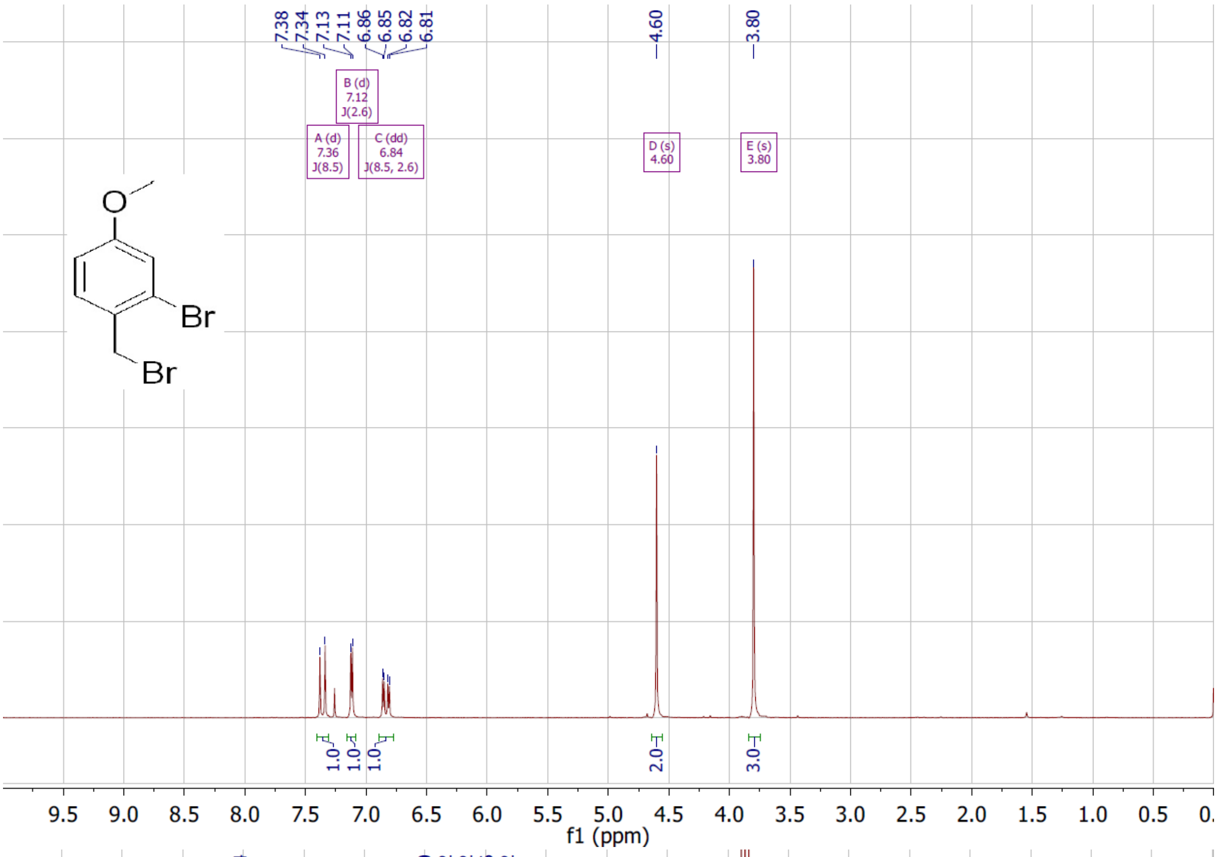
7.38
7.34
7.13
7.11
6.86
6.85
6.82
6.81
4.60
3.80
B (d) 7.12 J(2.6)
A (d) 7.36 J(8.5)
C (dd) 6.84 J(8.5, 2.6)
D (s) 4.60
E (s) 3.80
O
Br
Br
1.0
1.0
1.0
2.0
3.0
9.5 9.0 8.5 8.0 7.5 7.0 6.5 6.0 5.5 5.0 4.5 4.0 3.5 3.0 2.5 2.0 1.5 1.0 0.5
f1 (ppm)

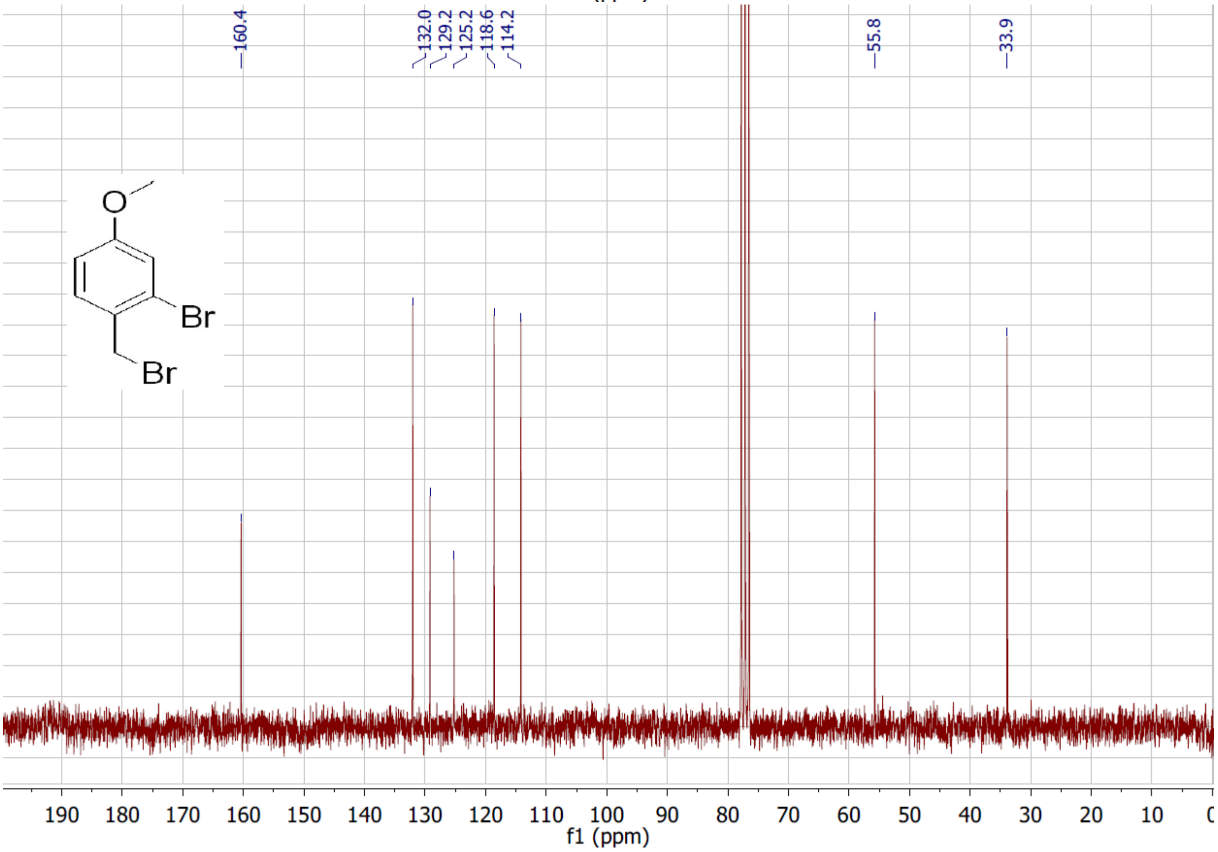
160.4
132.0
129.2
125.2
118.6
114.2
55.8
33.9
O
Br
Br
190 180 170 160 150 140 130 120 110 100 90 80 70 60 50 40 30 20 10
f1 (ppm)

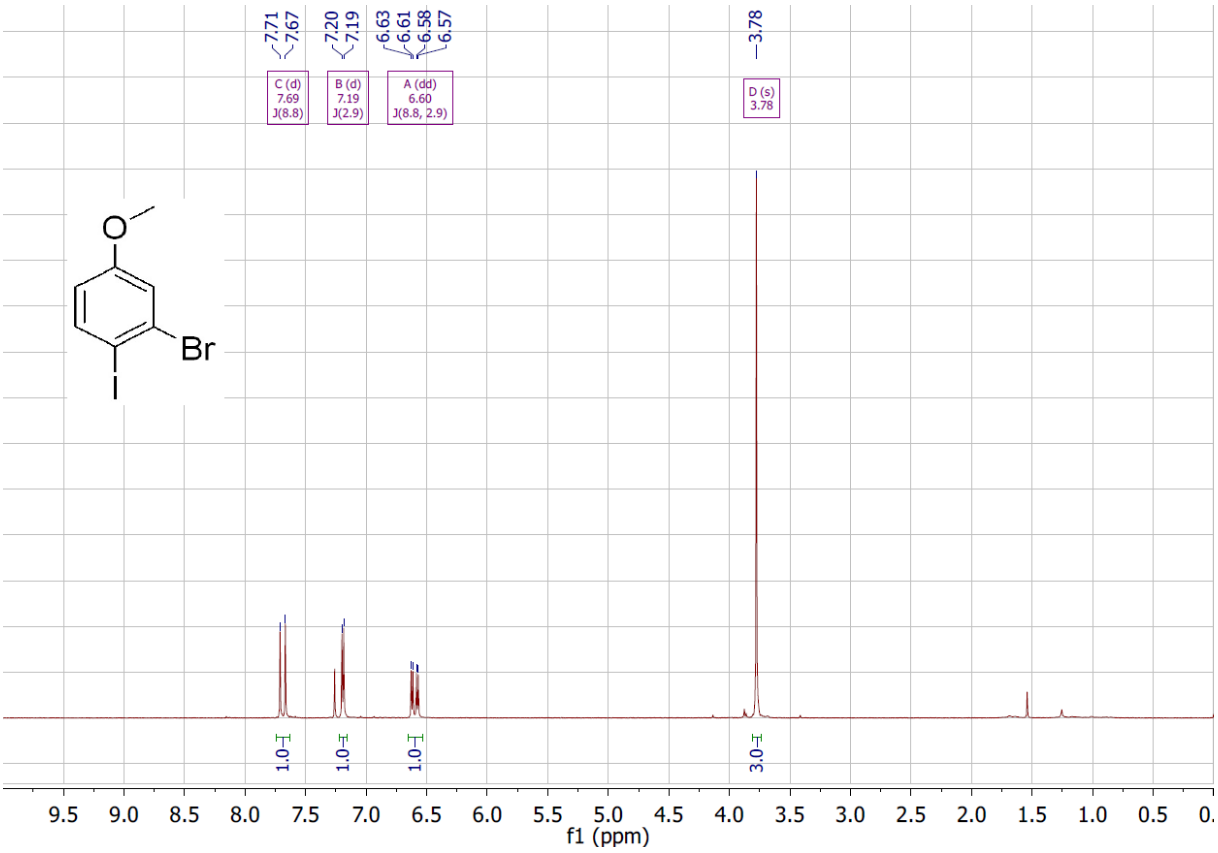
7.71
7.67
7.20
7.19
6.63
6.61
6.58
6.57
3.78
C (d) 7.69 J(8.8)
B (d) 7.19 J(2.9)
A (dd) 6.60 J(8.8, 2.9)
D (s) 3.78
O
Br
I
1.0
1.0
1.0
3.0
f1 (ppm)

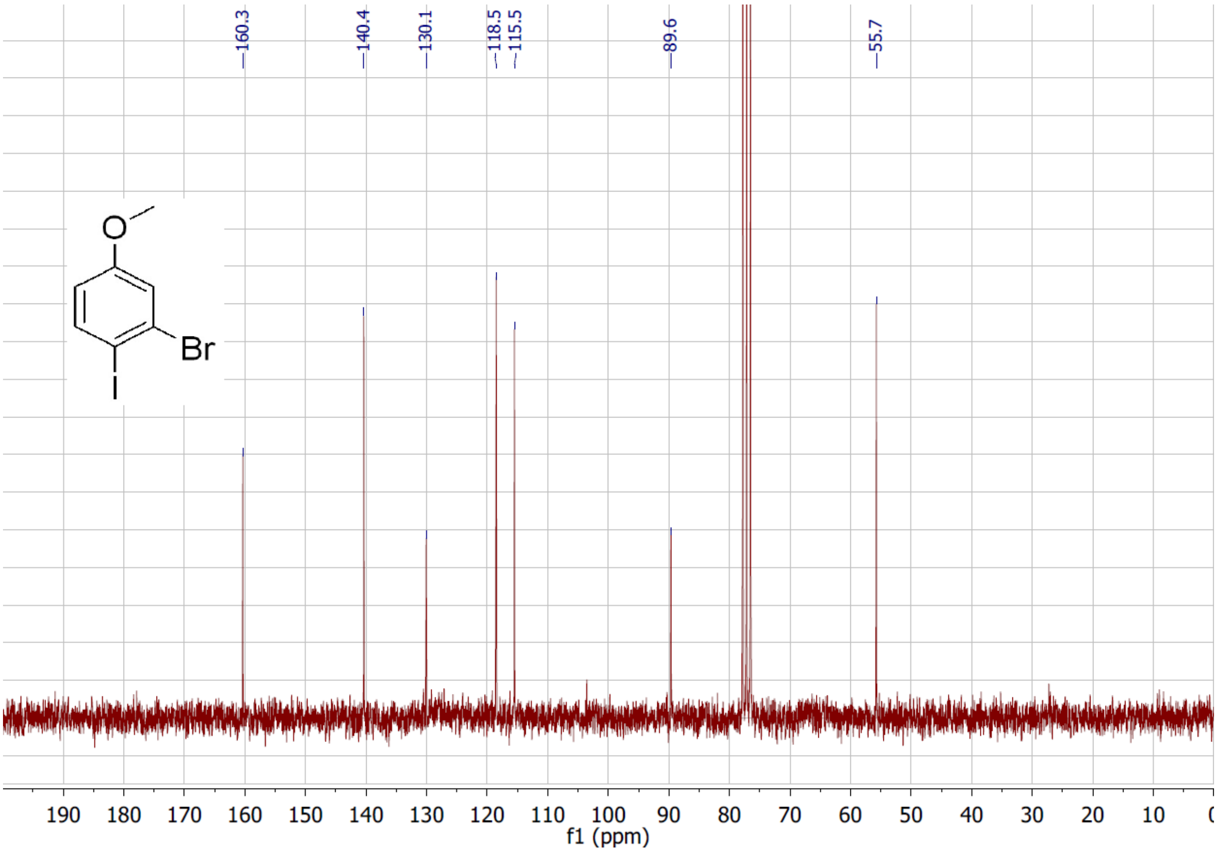
160.3
140.4
130.1
118.5
115.5
89.6
55.7
O
Br
I
f1 (ppm)

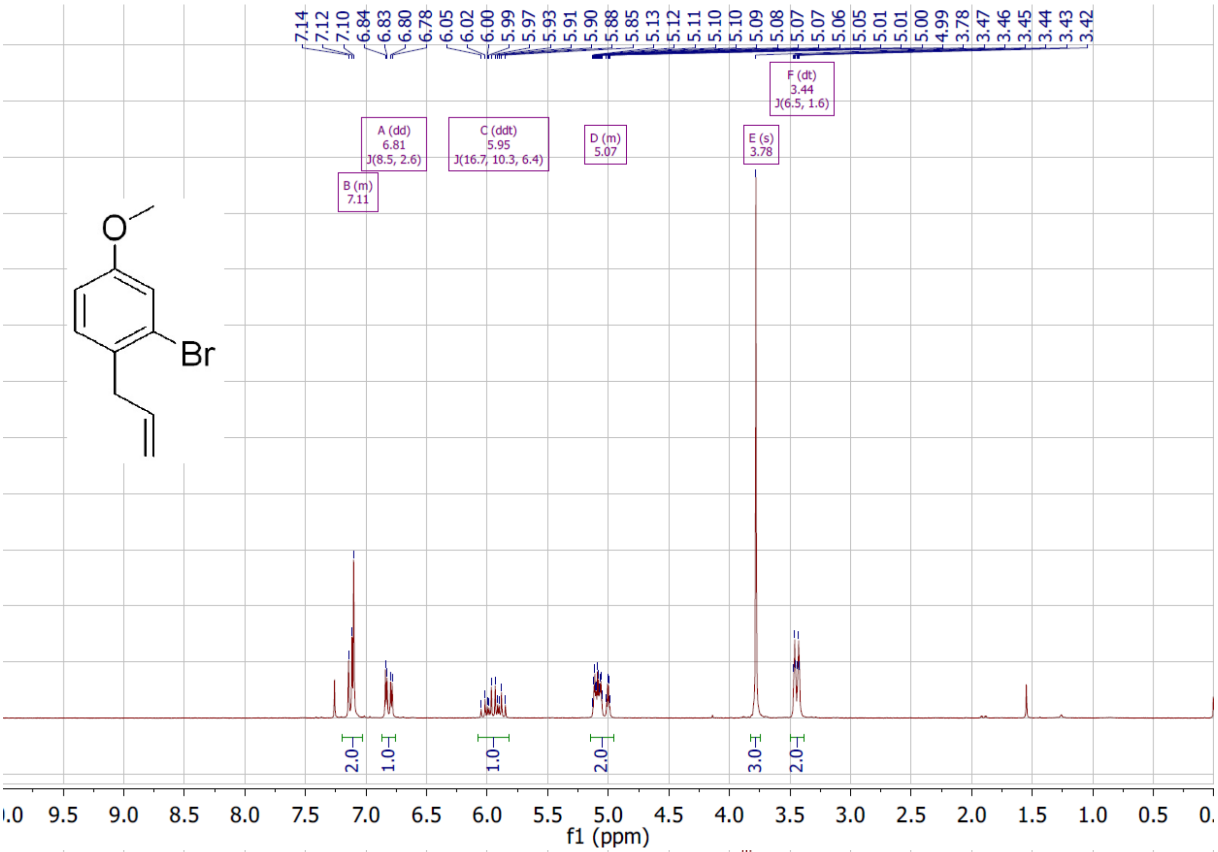
7.14 7.12 7.10 6.84 6.83 6.80 6.78 6.05 6.02 6.00 5.99 5.97 5.93 5.91 5.90 5.88 5.85 5.13 5.12 5.11 5.10 5.10 5.09 5.08 5.07 5.07 5.06 5.05 5.01 5.01 5.00 4.99 3.78 3.47 3.46 3.45 3.44 3.43 3.42
F (dt) 3.44 J(6.5, 1.6)
A (dd) 6.81 J(8.5, 2.6)
C (ddt) 5.95 J(16.7, 10.3, 6.4)
D (m) 5.07
E (s) 3.78
B (m) 7.11
O
Br
2.0
1.0
1.0
2.0
3.0
2.0
f1 (ppm)

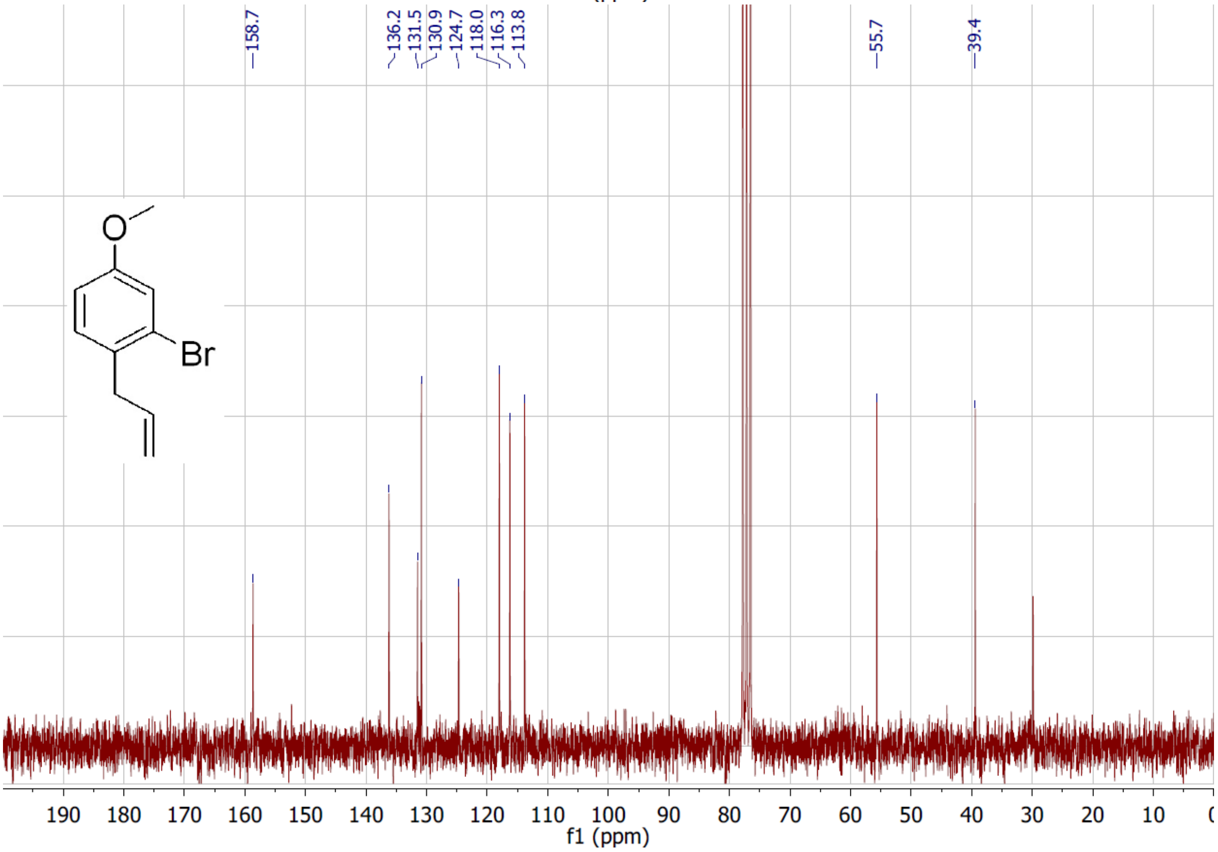
158.7
136.2 131.5 130.9 124.7 118.0 116.3 113.8
55.7
39.4
O
Br
f1 (ppm)

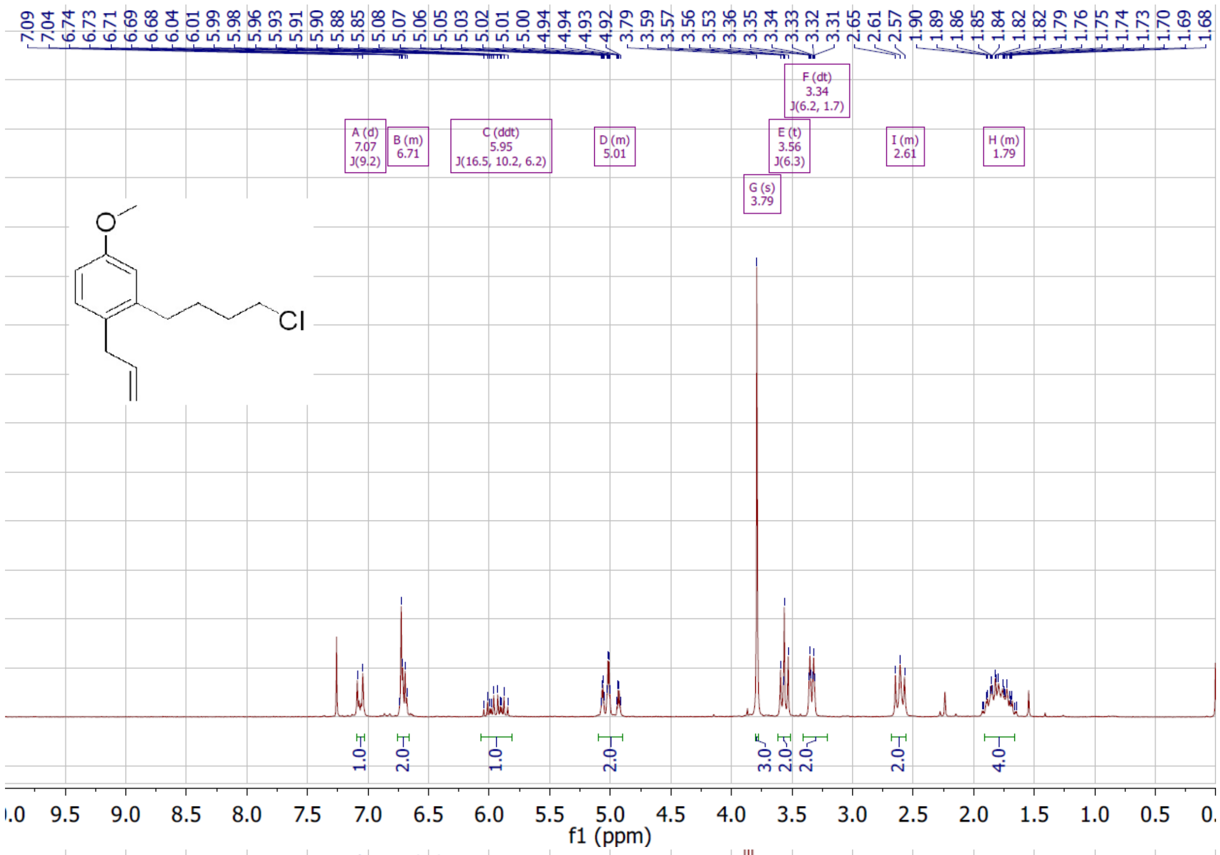
7.09 7.04 6.74 6.73 6.71 6.69 6.68 6.04 6.01 5.99 5.98 5.96 5.93 5.91 5.90 5.88 5.85 5.08 5.07 5.06 5.05 5.03 5.02 5.01 5.00 4.94 4.94 4.93 4.92 3.79 3.59 3.57 3.56 3.53 3.36 3.35 3.34 3.33 3.32 3.31 2.65 2.61 2.57 1.90 1.89 1.86 1.85 1.84 1.82 1.82 1.79 1.76 1.75 1.74 1.73 1.70 1.69 1.68
F (dt) 3.34 J(6.2, 1.7)
A (d) 7.07 J(9.2)
B (m) 6.71
C (ddt) 5.95 J(16.5, 10.2, 6.2)
D (m) 5.01
E (t) 3.56 J(6.3)
I (m) 2.61
H (m) 1.79
G (s) 3.79
O
Cl
1.0 2.0 1.0 2.0 3.0 2.0 2.0 2.0 4.0
f1 (ppm)

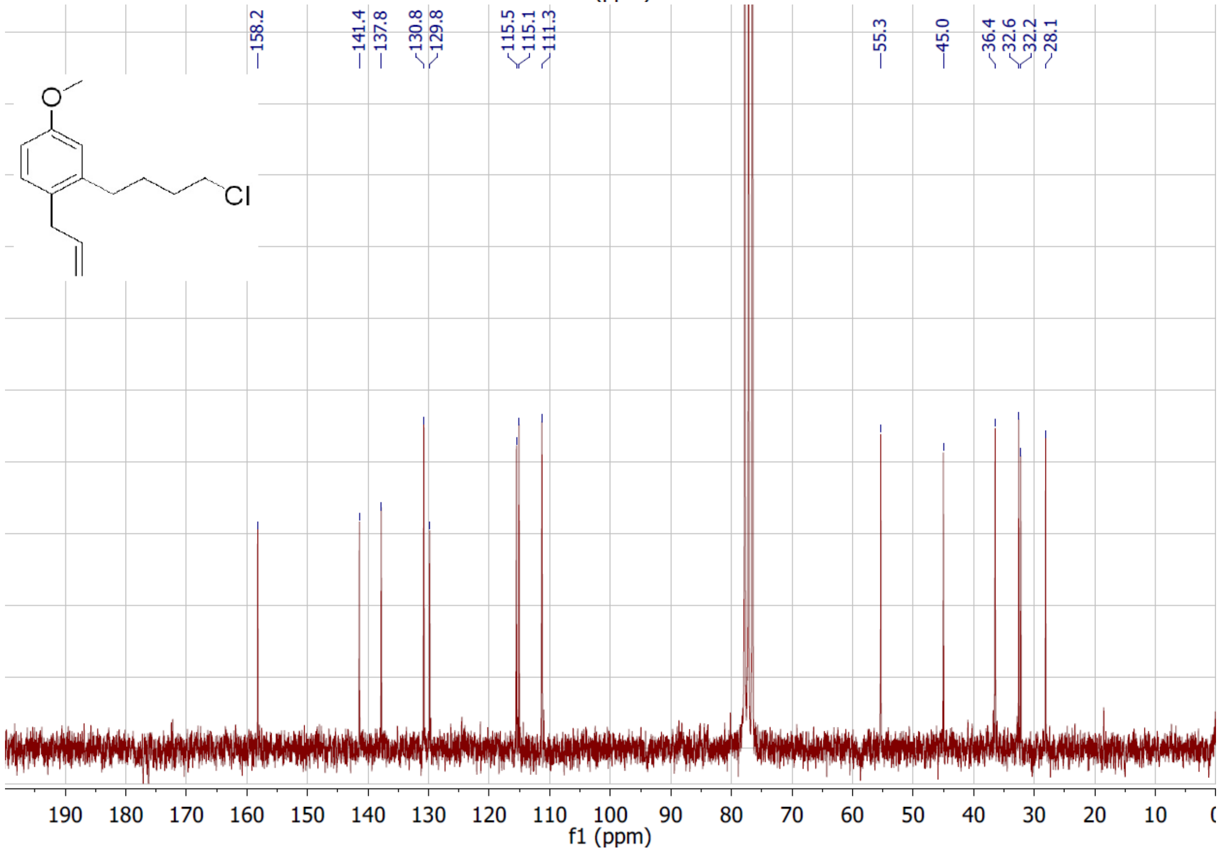
158.2 141.4 137.8 130.8 129.8 115.5 115.1 111.3 55.3 45.0 36.4 32.6 32.2 28.1
O
Cl
f1 (ppm)

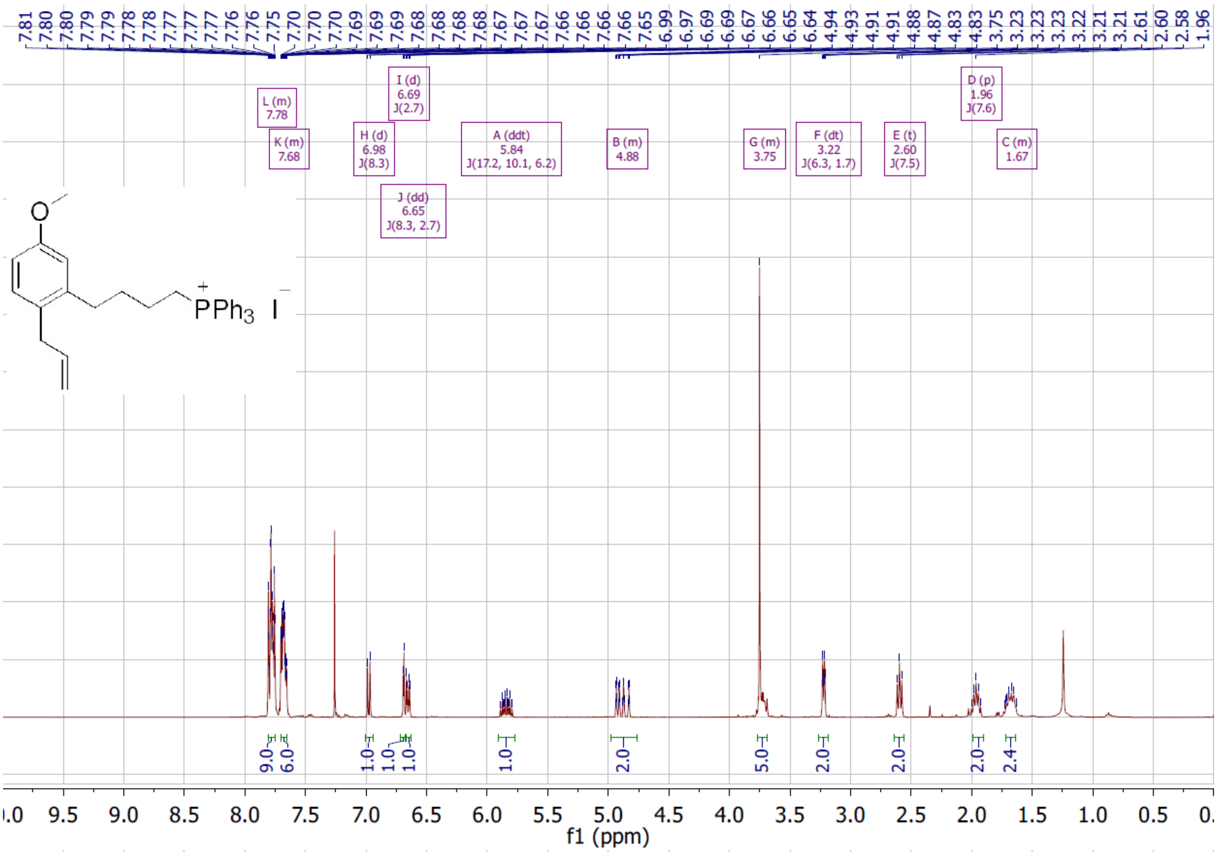
L (m) 7.78
K (m) 7.68
I (d) 6.69 J(2.7)
H (d) 6.98 J(8.3)
J (dd) 6.65 J(8.3, 2.7)
A (ddt) 5.84 J(17.2, 10.1, 6.2)
B (m) 4.88
G (m) 3.75
F (dt) 3.22 J(6.3, 1.7)
E (t) 2.60 J(7.5)
D (p) 1.96 J(7.6)
C (m) 1.67
PPh3 I
f1 (ppm)

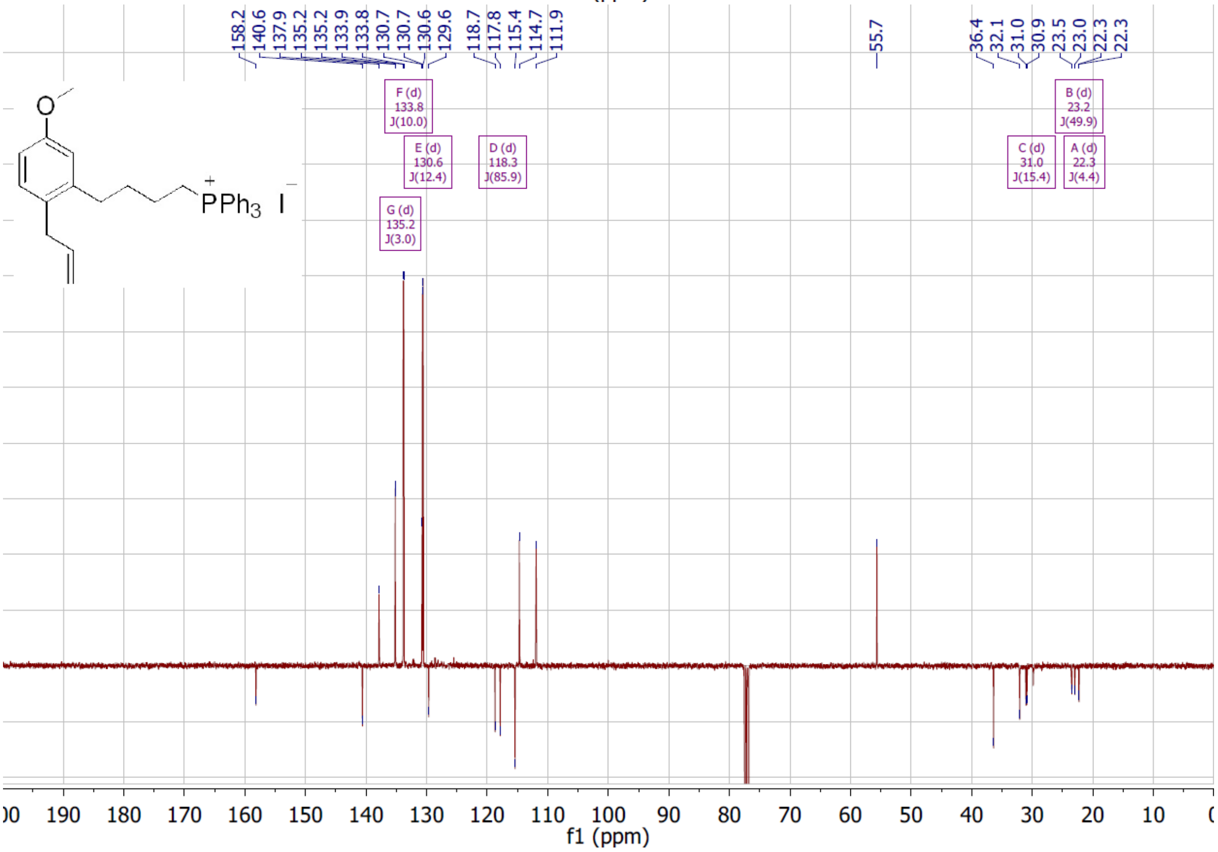
F (d) 133.8 J(10.0)
E (d) 130.6 J(12.4)
D (d) 118.3 J(85.9)
G (d) 135.2 J(3.0)
B (d) 23.2 J(49.9)
C (d) 31.0 J(15.4)
A (d) 22.3 J(4.4)
PPh3 I
f1 (ppm)

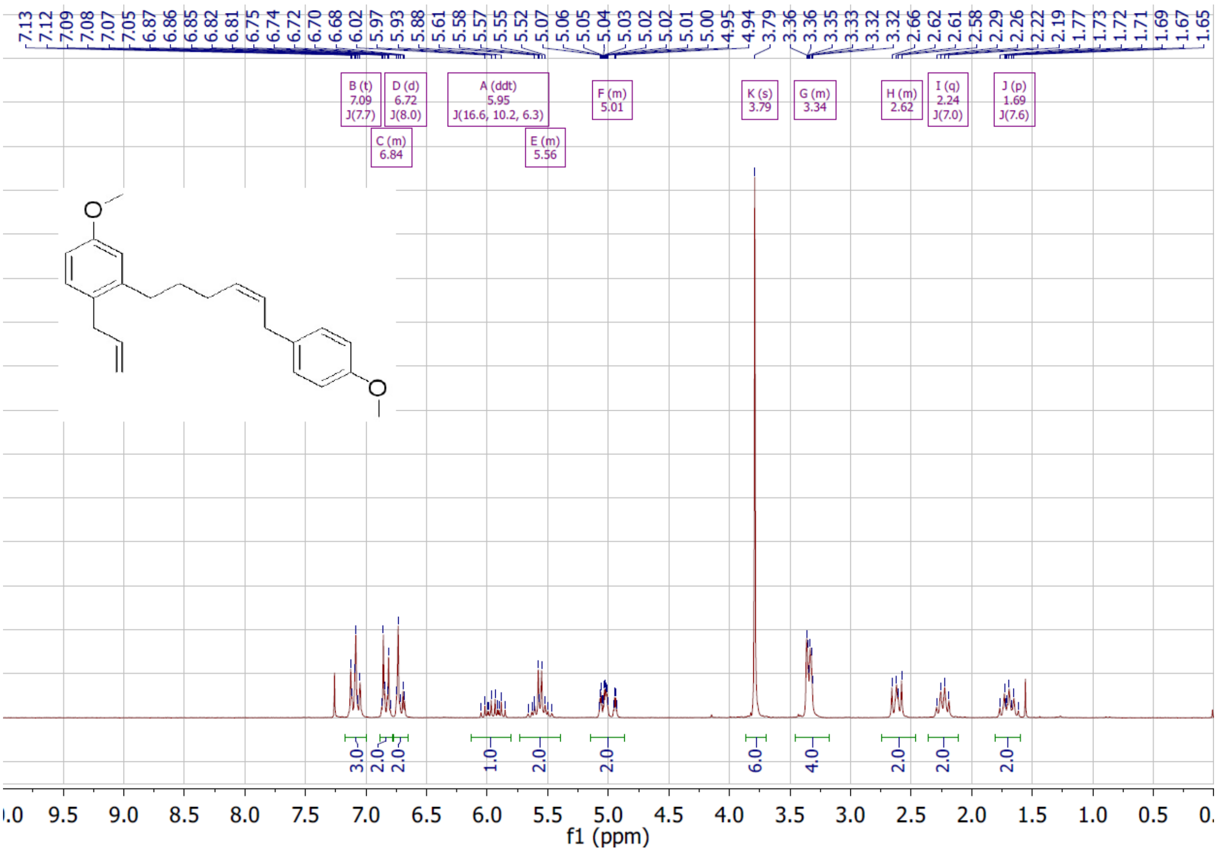
7.13 7.12 7.09 7.08 7.07 7.05 6.87 6.86 6.85 6.82 6.81 6.75 6.74 6.72 6.70 6.68 6.02 5.97 5.93 5.88 5.61 5.58 5.57 5.55 5.52 5.07 5.06 5.05 5.04 5.03 5.02 5.02 5.01 5.00 4.95 4.94 3.79 3.36 3.36 3.35 3.33 3.32 3.32 2.66 2.62 2.61 2.58 2.29 2.26 2.22 2.19 1.77 1.73 1.72 1.71 1.69 1.67 1.65
B (t) 7.09 J(7.7)
D (d) 6.72 J(8.0)
A (ddt) 5.95 J(16.6, 10.2, 6.3)
F (m) 5.01
K (s) 3.79
G (m) 3.34
H (m) 2.62
I (q) 2.24 J(7.0)
J (p) 1.69 J(7.6)
C (m) 6.84
E (m) 5.56
O
O
3.0 2.0 2.0 1.0 2.0 2.0 6.0 4.0 2.0 2.0 2.0
f1 (ppm)

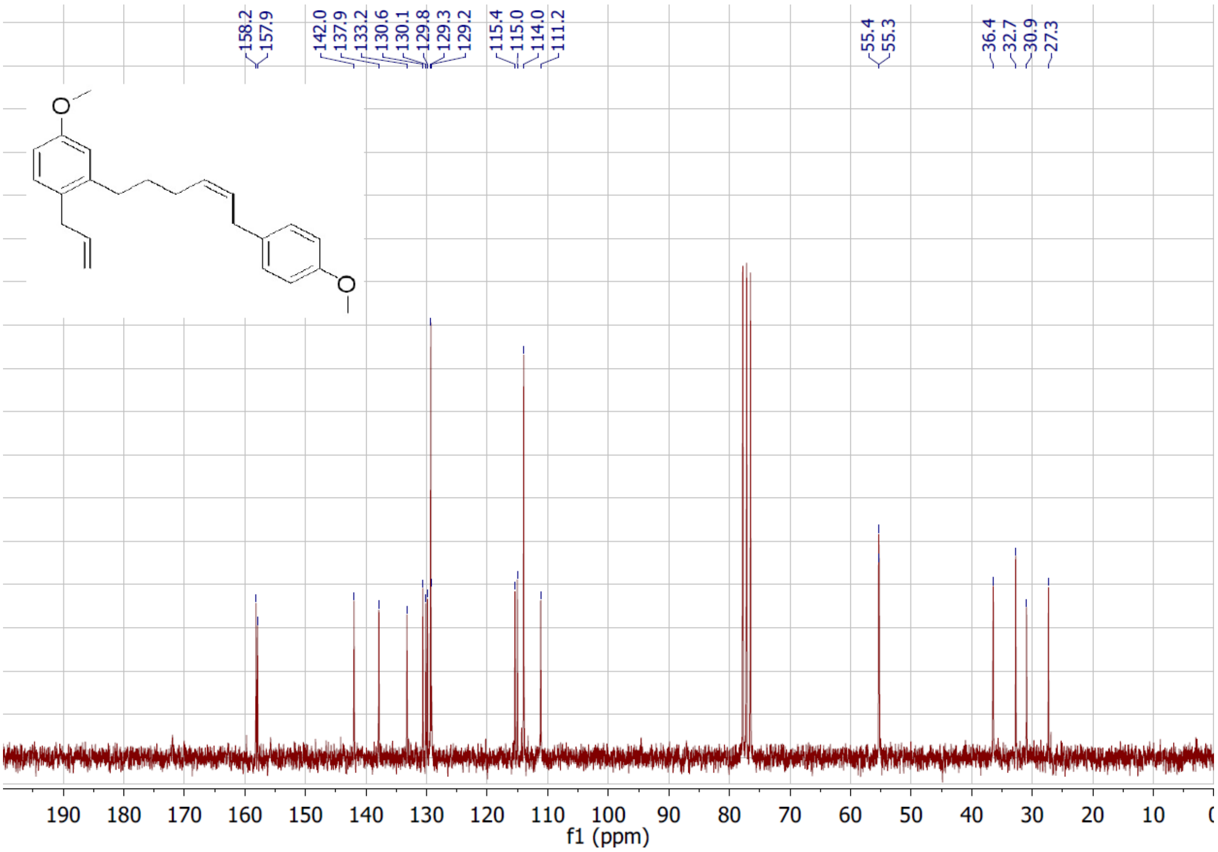
158.2 157.9 142.0 137.9 133.2 130.6 130.1 129.8 129.3 129.2 115.4 115.0 114.0 111.2 55.4 55.3 36.4 32.7 30.9 27.3
O
O
f1 (ppm)

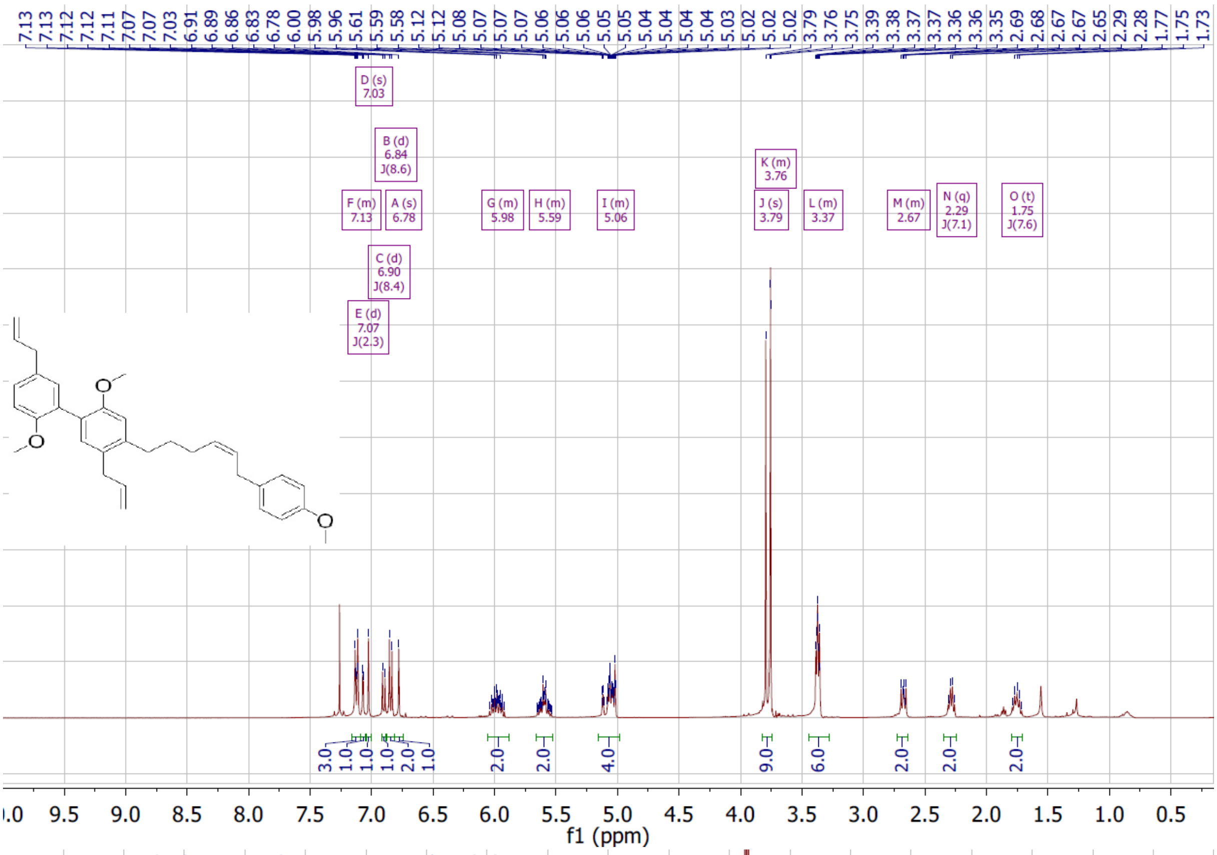
f1 (ppm)

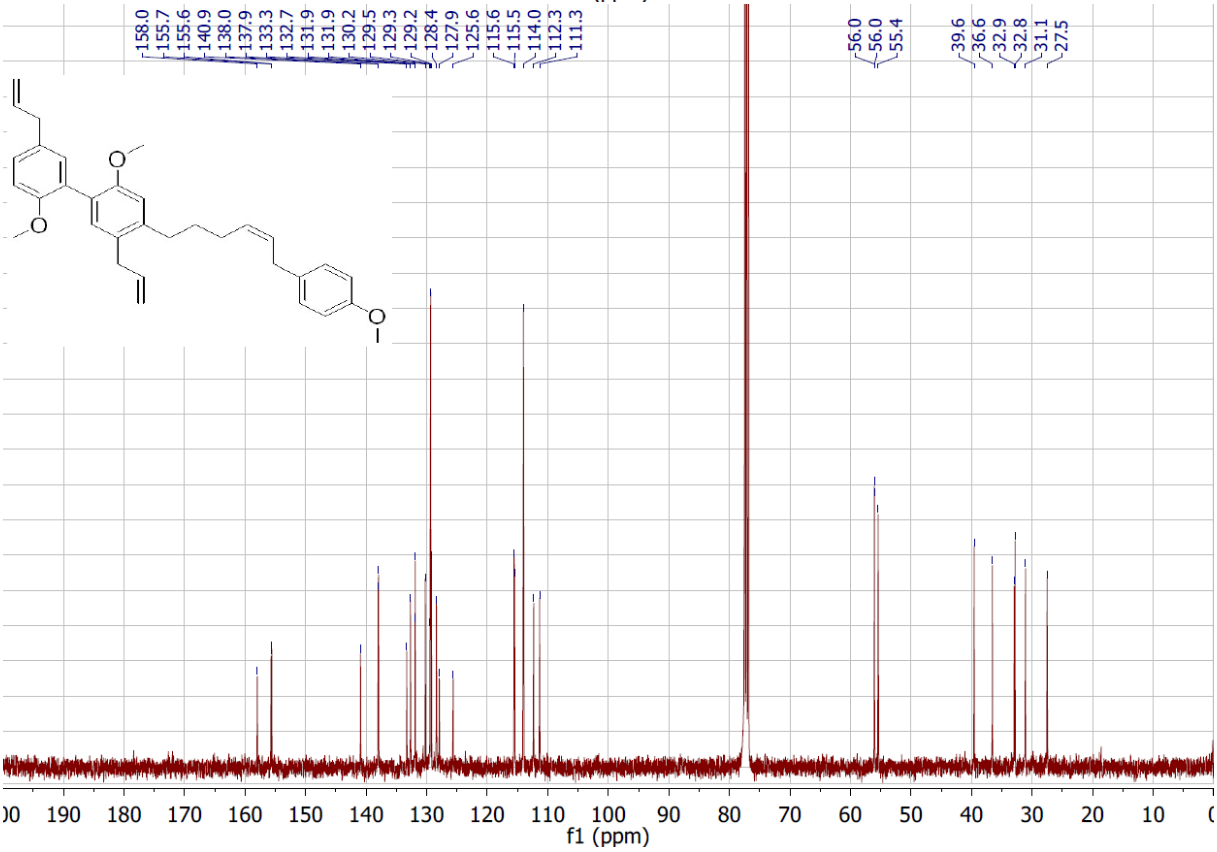
f1 (ppm)

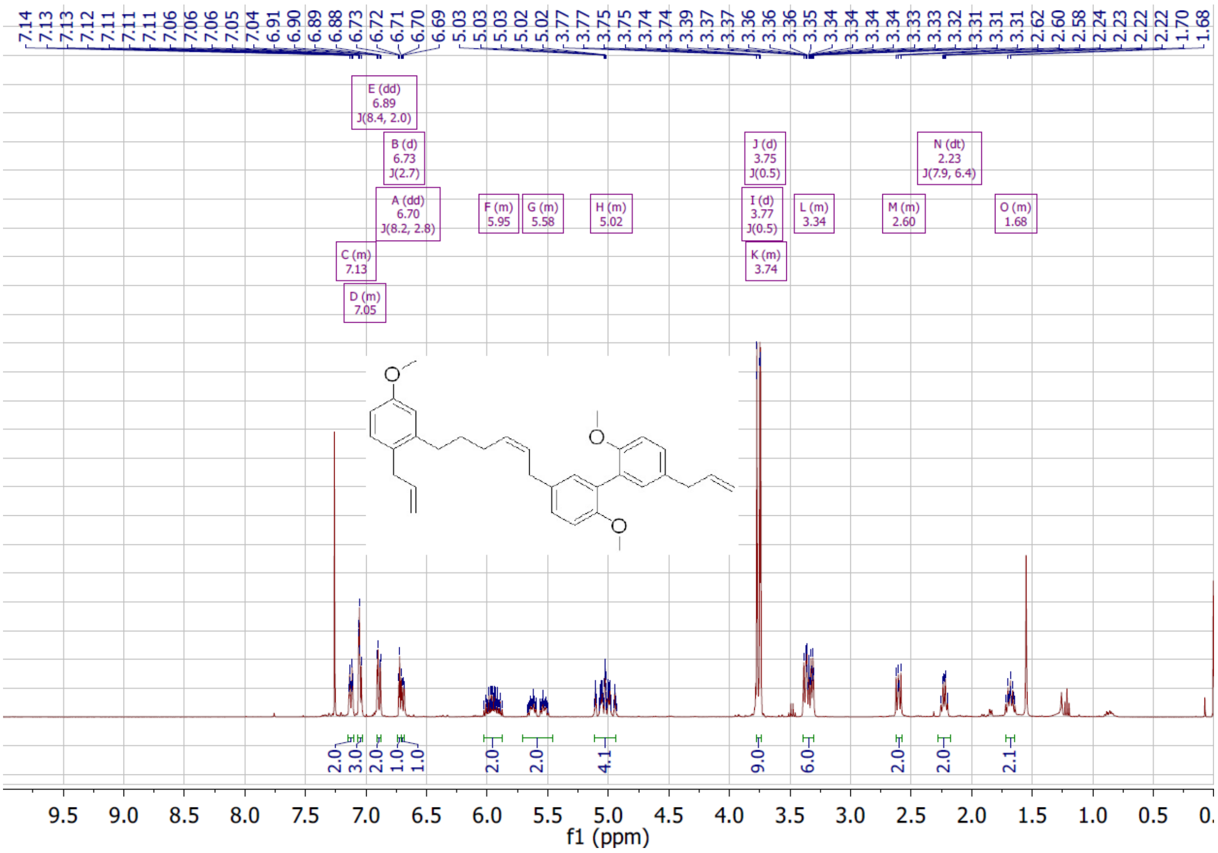
f1 (ppm)

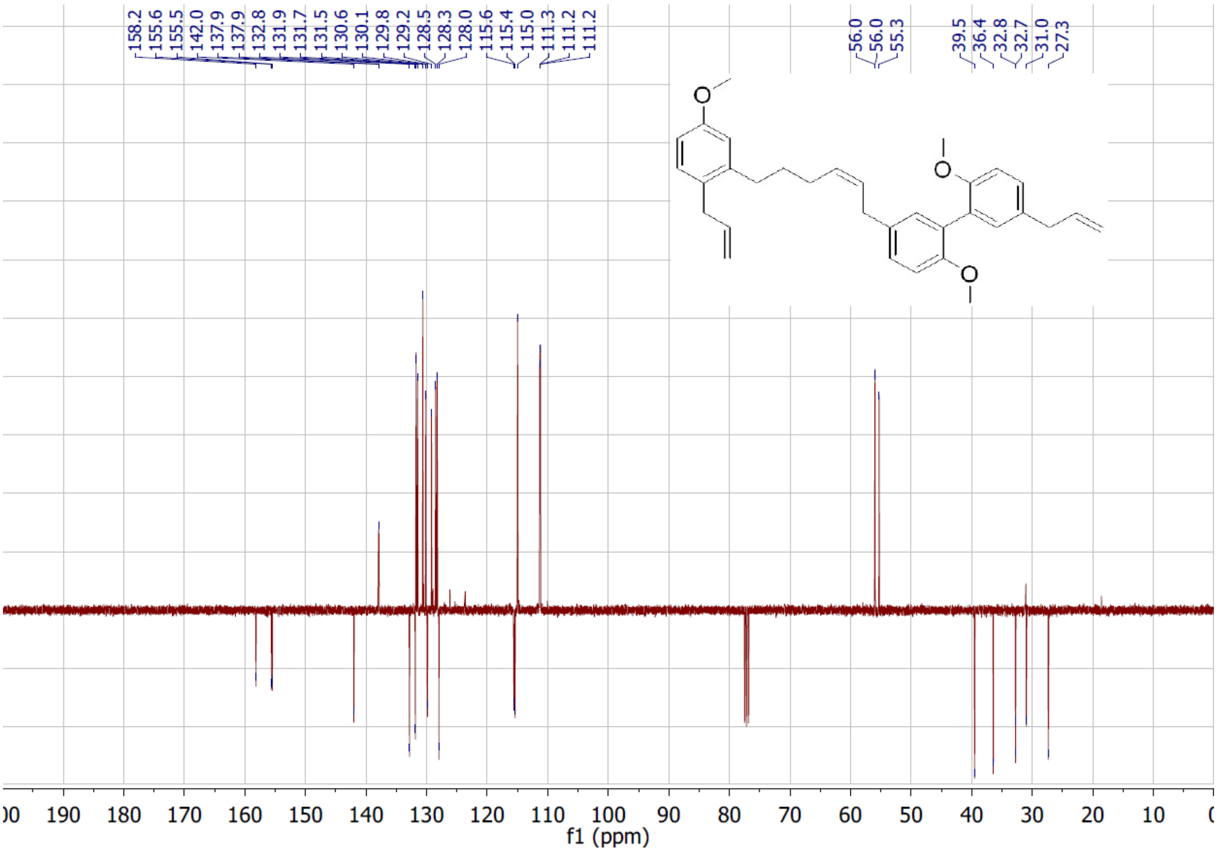
f1 (ppm)

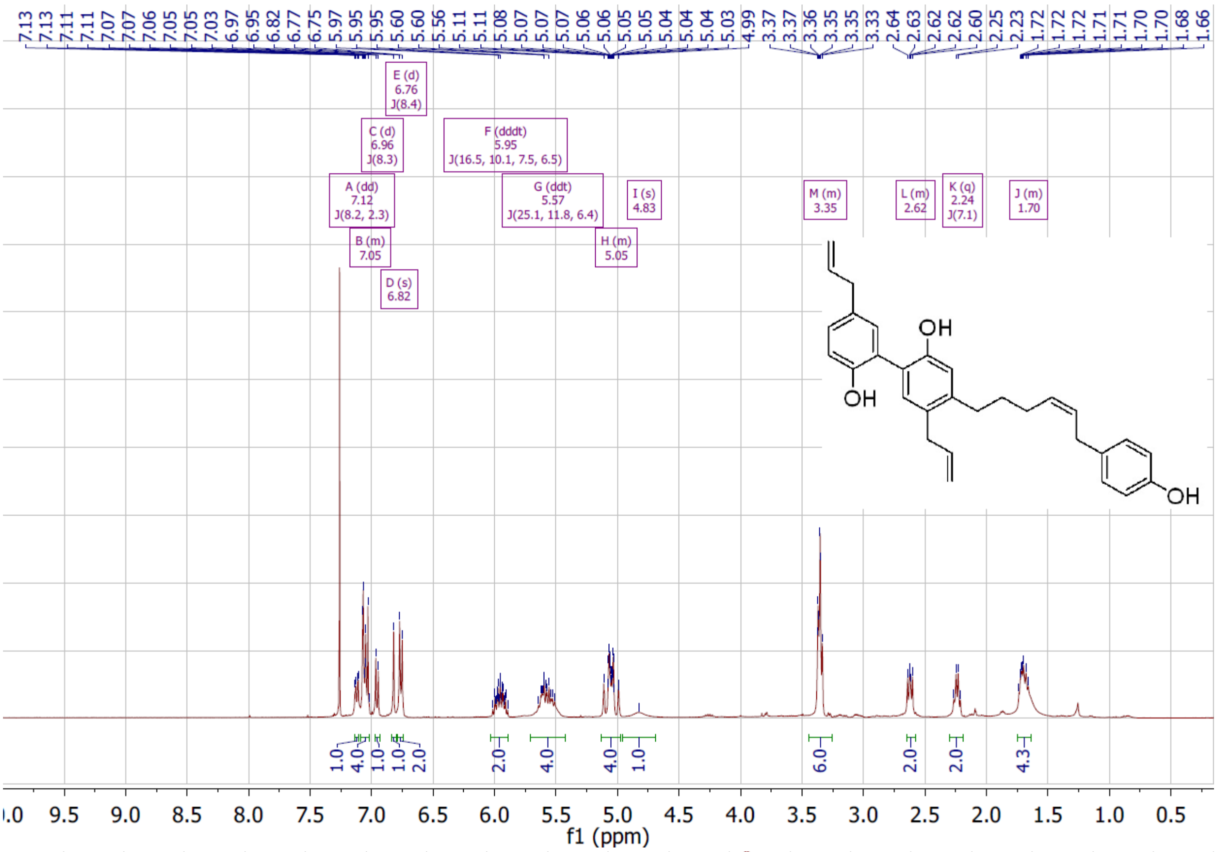
7.13 7.13 7.11 7.11 7.07 7.07 7.06 7.05 7.05 7.03 6.97 6.95 6.82 6.77 6.75 5.97 5.95 5.95 5.60 5.60 5.56 5.11 5.11 5.08 5.07 5.07 5.07 5.06 5.05 5.05 5.04 5.04 5.04 5.03 4.99 3.37 3.37 3.36 3.35 3.35 3.33 2.64 2.63 2.62 2.62 2.60 2.25 2.23 1.72 1.72 1.72 1.71 1.71 1.70 1.70 1.68 1.66
E (d) 6.76 J(8.4)
C (d) 6.96 J(8.3)
F (dddt) 5.95 J(16.5, 10.1, 7.5, 6.5)
A (dd) 7.12 J(8.2, 2.3)
G (ddt) 5.57 J(25.1, 11.8, 6.4)
I (s) 4.83
M (m) 3.35
L (m) 2.62
K (q) 2.24 J(7.1)
J (m) 1.70
B (m) 7.05
H (m) 5.05
D (s) 6.82
OH
OH
OH
1.0 4.0 1.0 1.0 2.0 2.0 4.0 4.0 1.0 6.0 2.0 2.0 4.3
f1 (ppm)

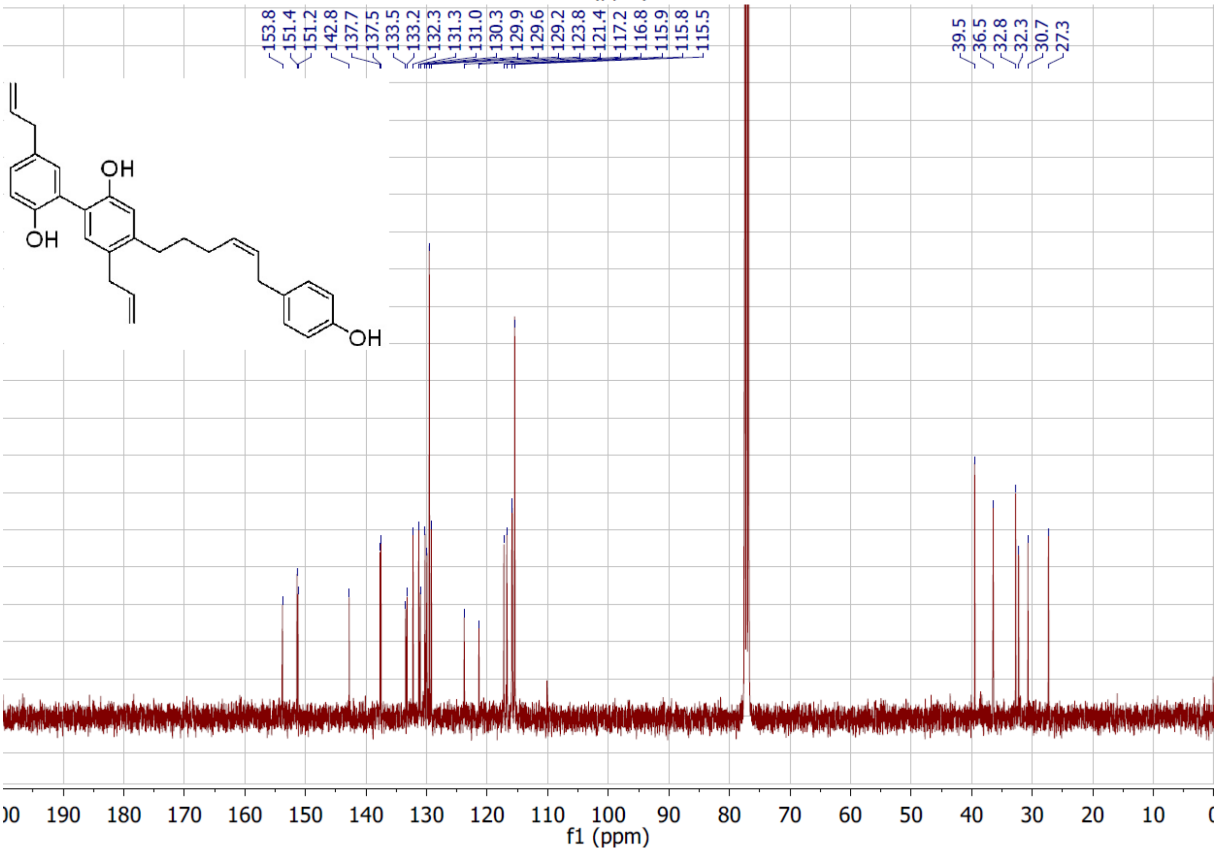
153.8 151.4 151.2 142.8 137.7 137.5 133.5 133.2 132.3 131.3 131.0 130.3 129.9 129.6 129.2 123.8 121.4 117.2 116.8 115.9 115.8 115.5
39.5 36.5 32.8 32.3 30.7 27.3
OH
OH
OH
f1 (ppm)

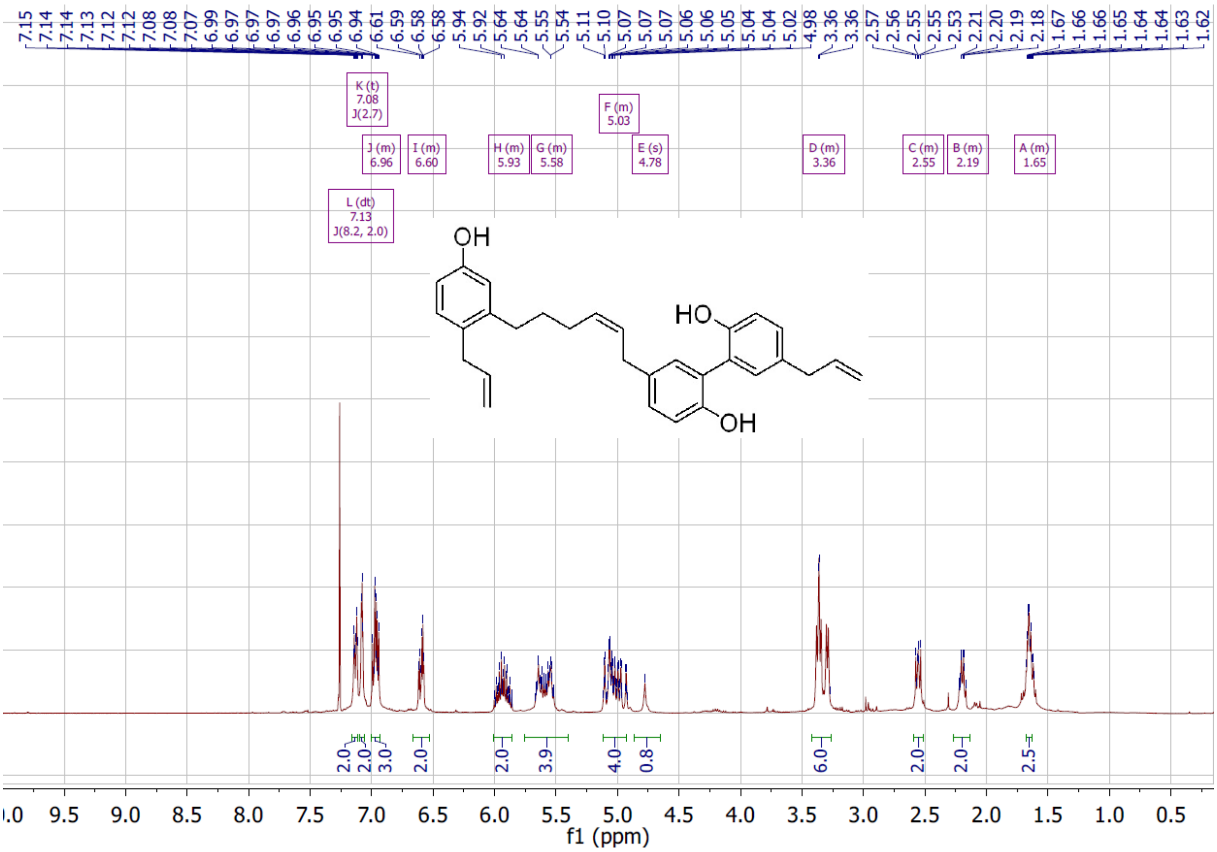

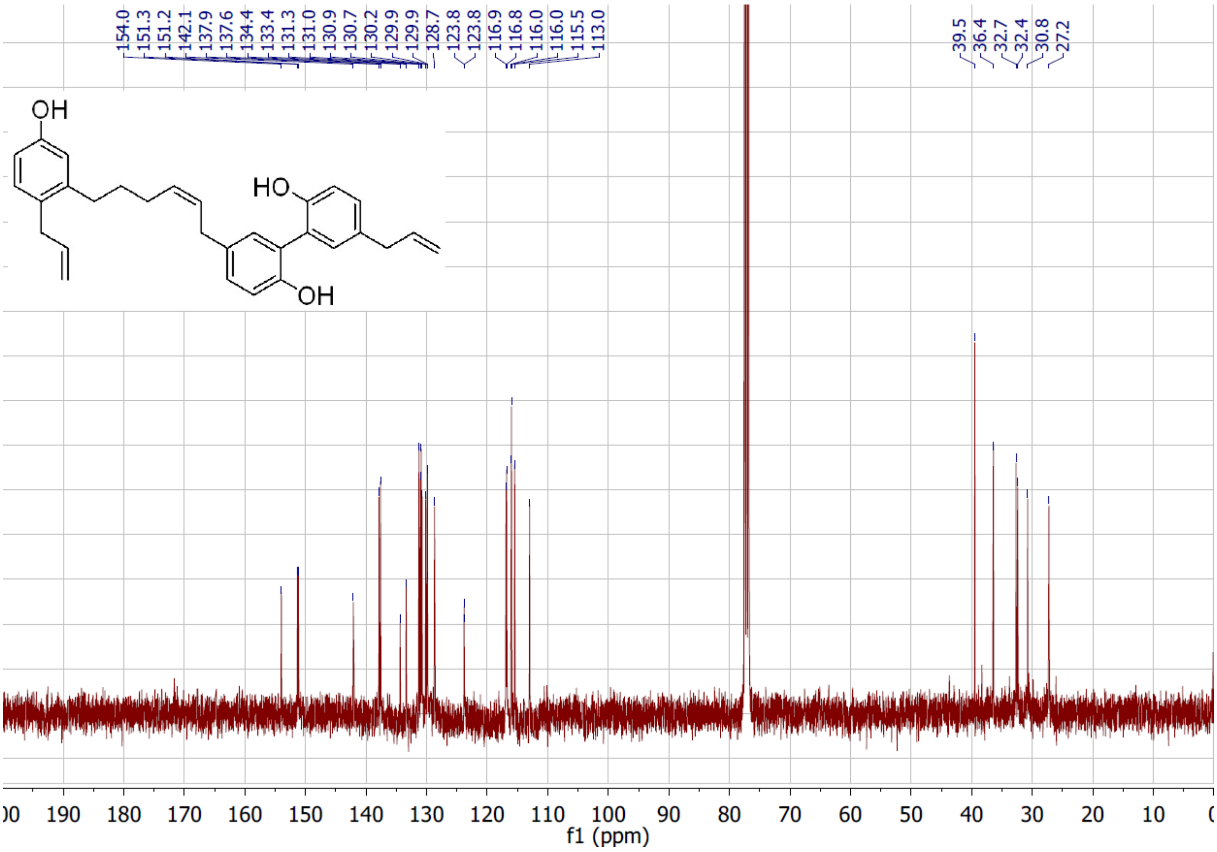

16

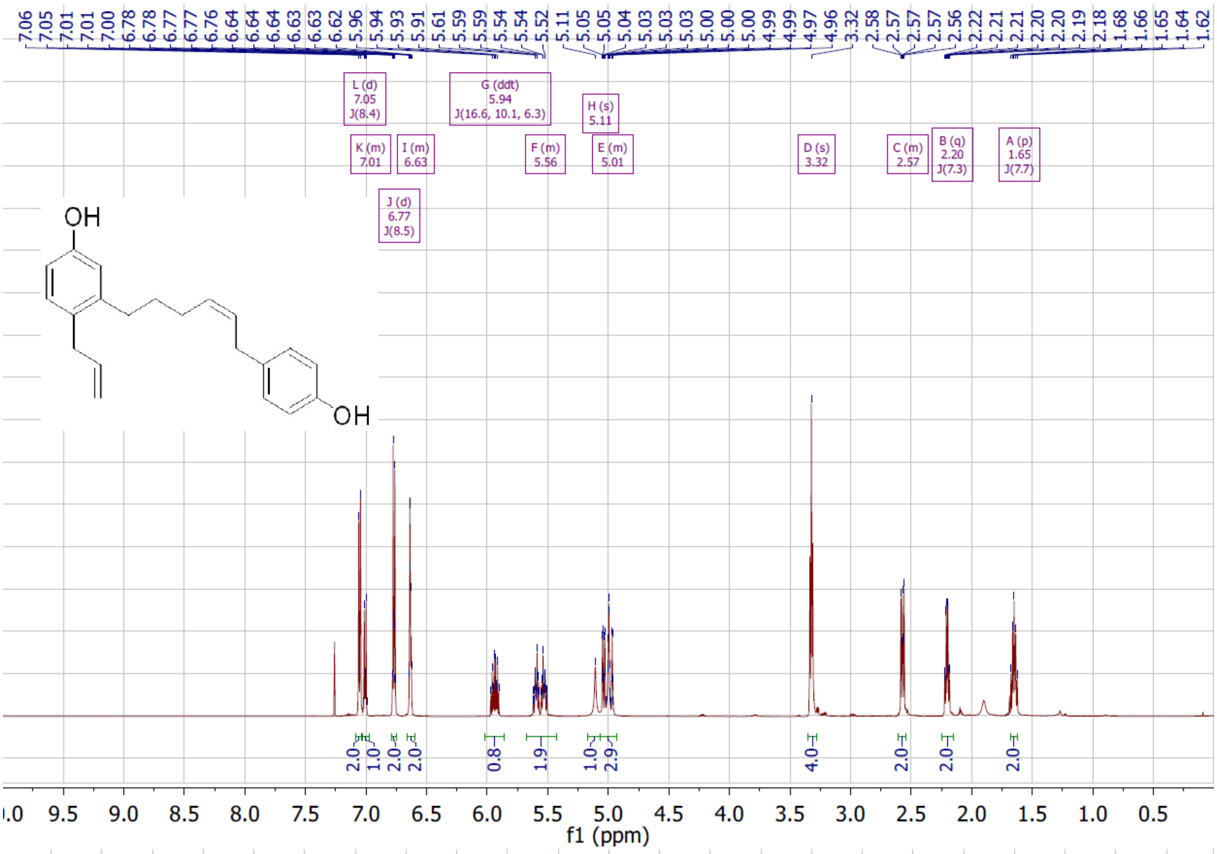

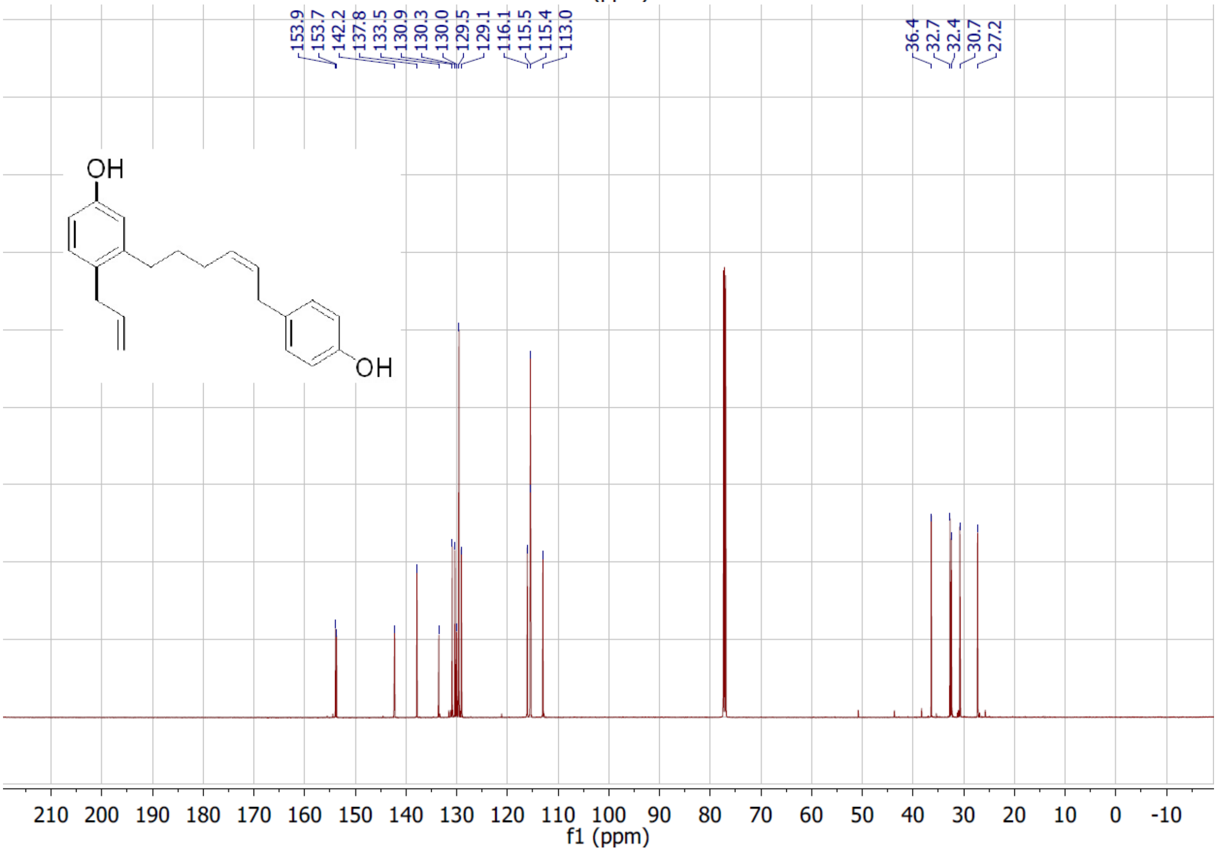

Supplement: Supplementary file 1 [file OB-016-C8OB01745J-s001.pdf]
